# TaqMan-MGB probe quantitative PCR assays to genotype and quantify three mtDNA mutations of Leber hereditary optic neuropathy

**DOI:** 10.1038/s41598-020-69220-7

**Published:** 2020-07-23

**Authors:** Bingqian Xue, Yang Li, Xin Wang, Rui Li, Xin Zeng, Meihua Yang, Xiaohui Xu, Tingbo Ye, Liming Bao, Yi Huang

**Affiliations:** 1 Chongqing Key Laboratory of Child Infection and Immunity, Ministry of Education Key Laboratory of Child Development and Disorders, National Clinical Research Center for Child Health and Disorders, China International Science and Technology Cooperation Base of Child Development and Critical Disorders, Children’s Hospital of Chongqing Medical University, No. 136 Zhongshan Erd Road, Yuzhong District, Chongqing, 400014 China; 2 Department of Laboratory Medicine, Zhengzhou Central Hospital Affiliated to Zhengzhou University, Zhengzhou , 450007 China; 3 Department of Neurosurgery, Xinqiao Hospital of Army Military Medical University, Chongqing, 400037 China; 4 The Third People’s Hospital of Chengdu, Chengdu, 610031 China; 5 Department of Pathology, University of Colorado School of Medicine, Aurora, CO 80045 USA

**Keywords:** Genetics, Neuroscience

## Abstract

Leber hereditary optic neuropathy (LHON) is a degenerative disease of the optic nerve associated with one of three mitochondrial DNA (mtDNA) m.3460G>A, m.11778G>A and m.14484T>C mutations. Although several procedures are available to genotype these mutations, quantitative approaches with rapid, low-cost and easy to handle advantages for three LHON mtDNA mutations are rarely reported. Here, we firstly developed a “one-step” tetra-primer amplification-refractory mutation system (T-ARMS) PCR for qualitative genotyping of three LHON mtDNA mutations. Subsequently, we established single, duplex and triplex TaqMan MGB probe-based fluorescence quantitative PCR (qPCR) assays to perform both qualitative and quantitative analyses of three LHON mtDNA mutations. Standard curves based on tenfold diluted plasmid standard exhibited high specificity and sensitivity, stable repeatability and reliable detectable ability of TaqMan probe qPCR assays without cross-reactivity upon probes combination. Moreover, by comparing with SYBR Green qPCR, we further validated the feasibility of the triplex-probe qPCR assay for the quantitative detection of mtDNA copy number in blood samples. In conclusion, our study describes a rapid, low-cost, easy to-handle, and high-throughput TaqMan-MGB probe qPCR assay to perform both qualitative and quantitative analysis of three primary LHON mtDNA mutations, offering a promising approach for genetic screening and testing of LHON mutations.

## Introduction

Leber hereditary optic neuropathy (LHON; MIM 535,000) is a maternally inherited mitochondrial DNA (mtDNA) disorder characterized by clinical presentations that include acute or sub-acute vision loss with a male prevalence^1,2^. Several mitochondrial mutations in genes coding subunits of NADH dehydrogenase (ND) are proposed to primarily be involved in disease development. The vast majority of cases are associated with one of three primary mtDNA mutations: m.3460G>A (MT-ND1 gene), m.11778 G>A (MT-ND4) and m.14484T>C (MT-ND6), which are responsible for over 95% of LHON cases worldwide^1^.

Genetically, LHON is characterized by a maternal inheritance pattern with a heteroplasmic mutation genotype and low penetrance^2^. A unique characteristic of mitochondrial mutations is the heteroplasmic state with co-existence of both wild-type (*Wt*) and mutant (*Mut*) mtDNA^3,4^. Owing to incomplete and variable penetrance, LHON mtDNA mutations lead to a considerable number of undetected, asymptomatic carriers with LHON mutations in the general population and their offspring are at increased risk of developing visual loss^5–7^. Notably, recent advances have also showed that heteroplasmic variety in these LHON mutations significantly correlates with disease severity^7^. Thus, precise quantification of heteroplasmic mtDNA mutations is particularly important for evaluating variable clinical phenotypes and providing genetic counselling for patients and families.

The conventional approaches in genotyping LHON mtDNA mutations are methods that use the PCR product of the DNA fragment containing the mutation, such as denaturing gradient gel electrophoresis(DDGE)^8,9^, single-stranded conformational polymorphism (SSCP)^10,11^, real-time SYBR Green PCR^12,13^, temporal temperature gradient gel electrophoresis^14^, denaturing high-performance liquid chromatography (DHPLC)^15^, solid-phase minisequencing^16^, fluorescence probe-based invader assay^17^ and PCR-restriction fragment length polymorphism (RFLP)^18^. Although these molecular tools for molecular screening of LHON have become popular, often they are expensive, time-consuming or difficult to handle, and sometimes challenging to reproduce or define precise heteroplasmic levels of LHON mutations. Therefore, developing a rapid, low cost and reliable assay with precise quantitation is essential to estimate heteroplasmic levels of LHON mtDNA mutations both in individuals and across family.

In the present study, we developed a “one-step” qualitative PCR technique based on the “Tetra-primer Amplification Refractory Mutation System” (T-ARMS) to define mutation types of three primary LHON mutations, which is suitable for routine pre-screening in conventional laboratories with limited resources. Following this approach, we established single, duplex and triplex TaqMan-minor groove binder (MGB) probe-based qPCR assays to perform both qualitative and quantitative analysis. Standard curves from plasmids containing a partial sequence of the mtDNA were used to obtain an absolute quantitation to test specificity, sensitivity, repeatability and detectability ratio of these qPCR assays. The feasibility of the triplex-probe qPCR assay was validated in 48 clinical blood samples. Our study describes a rapid, low cost, easy to handle and reliable assay for qualitative and quantitative analysis of three primary LHON mutations.

## Materials and methods

### Clinical samples and DNA isolation

A total of 48 human peripheral blood samples (45 samples from unaffected individuals and three samples from patients carrying the m.3460G>A, m.11778G>A and m.14484T>C mutations) were collected from the Children's Hospital of Chongqing Medical University. Three positive samples were confirmed to be heteroplasmic mutations by DNA sequencing. The study was approved by the Children's Hospital of Chongqing Medical University review board, and informed consent was obtained from all the participants. All blood samples were handled anonymously according to ethical standards and the Helsinki declaration. Total genomic DNA from peripheral blood was extracted using a TIANGEN Blood Genomic DNA Extraction Kit (Tiangen, Beijing, China), quantified by a NanoDrop Microvolume Spectrophotometers (Thermo Fisher, Waltham, MA, USA), and was diluted to 5 ng/μl in Easy dilution buffer (Takara, Dalian, China). All DNA samples were randomly ordered and double-blind tested.

### The plasmid DNA standard

For the standard template generation, *DNA* fragments containing three *Wt* LHON sites from total DNA were obtained by PCR amplification using outer control (OC) primers and then inserted into the PUCM-T T-A clone vector (Takara, Dalian, China) (Table 1). Homoplasmic mutation DNA was commercially constructed by the point mutation method (Sangon, Shanghai, China). The recombinant standard plasmids were purified by a Tiangen plasmid purification kit (Tiangen, Beijing, China) and measured by a NanoDrop Microvolume Spectrophotometers (Thermo Fisher, Waltham, MA, USA). The copy numbers of the plasmids were calculated according to the formula: Copy Number (Copies/μl) = Concentration (g/μl) / (660 × DNA length) × NA (NA: Avogadro's constant).

**Table 1:** Primers and amplification conditions of tetra-primer ARMS–PCR.

Genetic polymorphism	MT gene location	Primer sequence (5′–3′)	Tm (°C)	Touch-down PCR	Annealing Tm (°C)	Amplicon size (bp)
m..3460G>A	MT-ND6	F-inner primer (A allele):	69	No	65	217 (A allele)
		GCTACTACAACCCTTCGCTGCCA ^a^				
		R-inner primer (G allele):	67			292 (G allele)
		GGGCTCTTTGGTGAAGAGTTTTATTGC^a^				
		F-OC primer:	65			460 (OC)
		AGTATTATACCCACACCCACCCAAGAACA				
		R-OC primer:	64			
		GATTGAGTAAACGGCTAGGCTAGAGGTG				
m.11778G>A	MT-ND1	F-inner primer (A allele):	69	No	65	127 (A allele)
		CAAACTACGAACGCACTCACAGGCA^a^				
		R- inner primer (G allele) :	65			276 (G allele)
		TTGAAGTCCTTGAGAGAGGATTATGAGGC^a^				
		F-OC primer:	65			350 (OC)
		GCCTACCCCTTCCTTGTACTATCCCTATG				
		R-OC primer:	65			
		TTAATAGTGGGGGGTAAGGCGAGGTT				
m.14484T>C	MT-ND4	F-inner primer (A allele):	63	No	55	111 (T allele)
		CATCGCTGTAGTATATCCAAAGACAACAAT^a^				
		R-inner primer (G allele):	63			236 (C allele)
		AATAGTTTTTTTAATTTATTTAGGGGGAAGGG^a^				
		F-OC primer:	68			286 (OC)
		ACCCCTCTCCTTCATAAATTATTCAGCTT				
		R-OC primer:	63			
		GTGGTCGGGTGTGTTATTATTCTGAATT				

*MT* mitochondrial, *ND* NADH dehydrogenase, *F* forward, *R* reverse, *OC* outer control, *Tm* temperature.

^a^Underlined bases indicate mismatch base.

### Determination of mtDNA copy number in genomic DNA

The relative copy number of mtDNA in terms of genomic DNA from blood samples was determined by calculating the ratio of the human mitochondrial 12S ribosomal RNA (12 s rRNA) gene to the haemoglobin subunit beta gene (β-globin) using SYBR Green PCR as previously described^7,19^. The standard curves for 12 s rRNA and β-globin genes were constructed by PCR amplification production using SYBR Green dye (Toyobo, Shanghai, China). The copy number of the target genes was calculated using the following formula: log_10_(CN) = −log_10_ (1 + Eff) × CT + log_10_ (CN^T^) (CN: copy number; Eff: amplification efficiency; CN^T^: copy number detection threshold).The relative copy number of mtDNA in terms of genomic DNA was calculated by analyzing the copy number ratio of 12 s rRNA to β-globin. PCR primers and amplification conditions for 12 s rRNA and β-globin genes are listed in Supplemental Table 1.

### Qualitative T-ARMS-PCR assay

The T-ARMS PCR procedure was established to qualitatively genotype three LHON mtDNA mutations. Primers were optimized according to the human mtDNA sequence (GenBank Accession No. NC_012920.1) using web-based software made accessible by Ye et al.^20,21^. Primers and amplification conditions are listed in Table 4. PCR reaction was carried out within a PCR thermal cycler (Bio-Rad, Herculus, CA, USA) in a total volume of 25 μl containing 25–50 ng of DNA template, 12.5 μl 2× GoTaq Hot Start Green Master Mix (Promega, Madison, USA), 0.1 μM of each outer primer and 1.0 μM of each inner primer. PCR was commenced at 95 °C for 10 min, followed by 35 cycles of 30 s denaturation (95 °C), 30 s annealing (annealing Tms for different PCRs are described in Table 1), 30 s extension (72 °C) and an additional 5 min extension at 72 °C at the end of 35 cycles. A 5–10 μL aliquot of PCR products was loaded on 2.5% agarose gels with TAE buffer for separation by electrophoresis using the Alphalmager UV system (Bio-Techne, Minneapolis, MN, USA).

### TaqMan-MGB probe quantitative PCR assay

Specific primers and probes of the three LHON mtDNA mutations were designed for TaqMan MGB probe fluorescence qPCR based on human mtDNA sequences (GenBank accession no. NC_012920.1) using AlleleID 6.0 software and synthesized by Invitrogen (Shanghai, China) (Table 2). TaqMan MGB *Wt* and *Mut* probes were labelled with the fluorescent reporter dyes FAM, CY5 or NED at the 5′ end and the non-fluorescent quencher-minor groove binder (NFQ-MGB) at the 3′ end. The TaqMan-MGB probe qPCR was performed using the Premix Ex Taq™ Probe qPCR kit (Takara, Dalian, China) on the ABI 7,500 system (Thermo Fisher, USA) and analyzed by 7,500 Software v2.3. Single, duplex and triplex TaqMan-MGB probe qPCR were set up and were carried out in a 20-µl volume reaction containing 10 µl of supplied master mix and 5 ng of template DNA (Details are presented in Supplemental Table 2). The thermal cycling conditions were 1 min at 95 °C, followed by 40 cycles of 95 °C for 15 s, 62 °C for 40 s. The assay was repeated at least three times with each template.

**Table 2:** Primers, probes and amplification conditions of TaqMan-MGB probe qPCR.

Genetic polymorphism	MT gene location	Primer and probe sequences (5′–3′)	Tm (°C)	Annealing Tm (°C)	Amplicon size (bp)
m.3460G>A	MT-ND6	*Mut* probe (A allele):	68	62	179
		FAM-TTCGCTGACACCATA-MGB-NFQ ^a^			
		*Wt* probe (G allele):	68		
		NED-TTCGCTGACGCCATAA-MGB-NFQ ^a^			
		F-primer:	60		
		CAACCTCCTACTCCTCATTGTACC			
		R-primer:	60		
		CGGGTTTTAGGGGCTCTTTGG			
m.11778G>A	MT-ND1	*Mut* probe (A allele):	68	62	128
		CY5-CTCACAGTCACATCAT-MGB-NFQ ^a^			
		*Wt* probe (G allele):	68		
		FAM-CTCACAGTCGCATCAT-MGB-NFQ ^a^			
		F-primer:	60		
		CCTAGCAAACTCAAACTACGAACGC			
		R-primer:	60		
		GGGGTAAGGCGAGGTTAGCGA			
m.14484T>C	MT-ND4	*Mut* probe (C allele):	68	62	130
		NED-AAGACAACCACCATTC-MGB-NFQ ^a^			
		*Wt* probe (T allele):	68		
		CY5-AAGACAACCATCATTC-MGB-NFQ ^a^			
		F-primer:	60		
		CAGGATACTCCTCAATAGCCATCGC			
		R-primer:	60		
		GTGGTCGGGTGTGTTATTATTCTGA			

*MT* mitochondrial, *Mut* mutation, *Wt* Wild type, *ND* NADH dehydrogenase, *MGB* minor groove binder, *Tm* Temperature, *F* Forward, *R* Reverse, *FAM* 6-Carboxyfluorescein, *NED* Benzofluorotrichlorocarboxy-fluorescein, *CY5* Indodicarbocyanine, *NFQ* Non-fluorescent quencher.

^a^Underlined bases indicate mismatch base.

### Standard curve for the TaqMan-MGB probe qPCR Assay

Standard curves were generated using tenfold serial dilutions of *Wt* or *Mut* plasmid DNA from 10^2^ to 10^8^ copies/reaction in 20-μL reactions. Five dilutions from 10^3^ to 10^7^ copies/reaction with a similar CT interval were used to construct standard curves by plotting the logarithm of the plasmid quantity against the measured CT values. The logarithm of the quantity was the abscissa and the CT value was used as the ordinate. By measuring the amplification CT value of the target probe in an unknown sample, the copy number of the corresponding mtDNA can be determined.

### Specificity and sensitivity of the TaqMan-MGB probe qPCR

The specificity of the TaqMan-MGB qPCR was determined using three different templates under the conditions as described earlier. *Wt* DNA templates (a mixture of three *Wt* plasmid templates at ratio 1:1:1), *Mut* DNA templates (a mixture of three *Mut* plasmid templates at ratio 1:1:1) and mixed *Wt* and *Mut* templates (a mixture of all six plasmid templates) were used. All templates were 10^8^ copies and reactions were performed in triplicate. The relative sensitivity of the TaqMan qPCR assay was tested for single-probe, duplex-probe, and triplex-probe assays by using tenfold standard dilutions ranging from 10^2^ to 10^8^ copies/reaction. Three replicates of each dilution and two negative blank controls were used.

### Repeatability and the detectable ratio of the TaqMan-MGB probe qPCR assay

The repeatability of the TaqMan MGB probe qPCR assay was estimated from the mean CT and standard deviation (SD) values obtained from five tenfold dilutions of the DNA standard with 10^3^ to 10^7^ copies per reaction of template in three replicates during a single reaction. The detectable ability of the qPCR assays was assessed by analyzing the template composed of all LHON plasmids with five different ratios of *Wt* or *Mut* template to mixed template (100/100, 75/100, and 25/100–1/100). All templates were 10^8^ copies and reactions were performed in triplicate.

### Assessment of qPCR to distinguish the different content of the copy number of mtDNA in genomic DNA

The capability of the triplex-probe TaqMan qPCR assays to distinguish heteroplasmic level was determined by detecting the copy number of mtDNA mutants in total DNA as previously described^7,22^. The mutation contents were divided into three levels: 80–100% for high dominant risk of vision loss, 25–80% for medium dominant risk of vision loss and 0–25% for low dominant risk of vision loss. Different mutation content (0–25% for three samples, 25–80% for 10 samples, 80–95% for three samples) were obtained by mixing the *Mut* plasmid templates and genomic DNA in proportion. The copy number of *Wt* and *Mut* mtDNA in mixed samples was calculated basing on the CT value and the standard curve.

## Results

### Establishment of qualitative T-ARMS-PCR for three LHON primary mutations

To rapidly genotype three primary LHON mutations, a “one-step” T-ARMS-PCR system was established by a series of optimized programs. As shown in the Supplemental Fig. 1A, four specific primers after NCBI blast specific analysis were employed to detect a single mutation hotspot. Two outer primers were utilized to amplify outer control fragment of the mutant hotspots while two inner primers were employed to specifically target *Wt* or *Mut* mutations by specific complementary of 3′ end of inner primers to the mutation site. An additional mismatch was introduced at the 1st or 3rd position upstream the 3′ end of the two allele-specific primers to increase their binding specificity to either *Wt* or *Mut* sequence targets (Table 1). All primers were optimized to reach close annealing temperatures and the size of allele-specific bands was determined by gel electrophoresis (Table 1 and Supplemental Fig. 1B–1C). Although touch-down PCR is desirable in T-ARMS PCR, our study showed that constant annealing temperature and an optimized 1:10 ratio of the outer versus inner primers could achieve high-quality and easily distinguishable products for the three homoplasmic and heteroplasmic LHON mutations, as well as well-balanced PCR product yields among outer control and inner allele-specific products that were further confirmed by DNA sequencing (Supplemental Fig. 2A–2C).

### Sensitivity analysis of T-ARMS-PCR

The ability to detect low levels of mtDNA mutations of the T-ARMS-PCR assay was further evaluated by titrating *Wt* and *Mut* standard DNA with decreasing DNA amounts from 200 × 10^9^ to 0.5 × 10^9^ copies (Supplemental Fig. 3A–3B). The results indicated that the assay produced reliable and reproducible amplification when the input DNA template was at 1.0 × 10^9^ copies (Supplemental Fig. 3A–3B). To evaluate the sensitivity of the T-ARMS-PCR to detect low proportions of heteroplasmic *Mut* DNA, a serial of DNA mixtures was generated by mixing genomic DNA containing known amounts of *Wt* mtDNA with *Mut* standard plasmid, and the ratios of *Wt to Mut were* from 1:2 to 1:200. We found that the *Mut* alleles were easily distinguished at the range of 1:50–1:2 dilution using mixtures DNA templates, although the product at a dilution of 1:100 was faintly visible (Supplemental Fig. 3C), indicating that the T-ARMS-PCR could distinguish as low as 1/50 *Mut* DNA from mixed templates. Thus, these results suggest that this single-step T-ARMS-PCR assay presents reasonable specificity and sensitivity for the qualitative detection of three LHON mtDNA mutations.

### Single TaqMan-MGB probe qPCR

**Figure 1:**
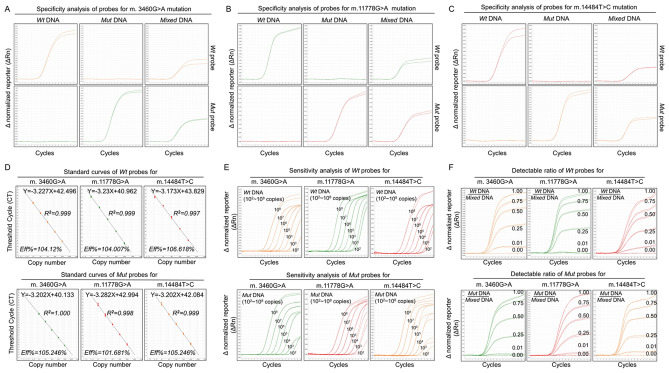
Single TaqMan-MGB Probe qPCR for Three LHON mtDNA Mutations. Single TaqMan-MGB probe qPCR was established using six single probes (three *Wt* probes & three *Mut* probes) targeting corresponding mtDNA mutations to independently detect *Wt*, *Mut* or mixed DNA template of three mtDNA mutations in six independent PCR reactions. (**A**–**C**) Specificity analysis of single-probe qPCR. Single *Wt* or *Mut* probe was used to detect corresponding DNA templates (*Wt*, *Mut* or mixed templates) from mtDNA mutations m.3460G>A (**A**), m.11778G>A (**B**) and m.14484T>C (**C**). (**D**) Standard curves of single-probe qPCR. Five tenfold dilutions (10^3^–10^7^ copies) were used to construct the standard curves in six independent PCR reactions by plotting the logarithm of the plasmid quantity against the measured CT values. Representative standard curves, Eff% and R^2^ are shown. (**E**) Sensitivity analysis of single-probe qPCR. The sensitivity of this qPCR was determined by detecting the limitation rank of the system with a tenfold dilution limitation ranking from 10^2^ to 10^8^ copies in six independent PCR reactions. Three replicates of each dilution were used. (**F**) Detectable ratio analysis of single-probe qPCR. The detectable ratio was assessed using DNA plasmid templates with five different ratios of *Wt* or *Mut* templates to mixed templates (100/100, 75/100, and 25/100 to 1/100). All template quantities were 10^8^ copies and the experiments were performed in triplicate. Representative curves were shown. *Wt* Wild type, *Mut* mutation, *Eff%* Efficiencies, *R*^2^ Coefficients of determination.

To further determine the copy number of mtDNA mutations in genomic DNA, we sought to establish a qualitative and quantitative TaqMan-MGB probe qPCR assay. Thus, we firstly designed a single-probe qPCR in which two independent *Wt* or *Mut* probes were designed to specifically target corresponding *Wt* or *Mut* allele for the same mtDNA mutation (Table 2). Specificity analysis showed that these *Wt* or *Mut* probes could specifically detect their corresponding targeting DNA even in the mixed templates containing three LHON mtDNA mutations (Fig. 1A–C), indicating that the single-probe qPCR could qualitatively distinguish three LHON mtDNA mutations from *Wt* DNA.

To assess the ability of this qPCR to precisely define the copy number of mtDNA in genomic DNA, we then generated six independent standard curves by a tenfold dilution experiment using six probes of three mtDNA mutations. In these curves, the coefficients of determination (R^2^) ranged from 0.997 to 1.000, and the calculated amplification efficiencies (Eff%) were within the range of 101.681–106.618% (Fig. 1D), indicating excellent linear correlations between CT values and DNA loading copy numbers. Next, we tested the sensitivity of this single-probe qPCR by tenfold serial dilutions from 10^2^ to 10^8^ copies/reaction and found that all these probes could detect DNA templates as low as 10^2^ copies/reaction (Fig. 1E), indicating the high sensitivity of this qPCR. Based on the results obtained from each dilution of standard plasmid DNA in five different experiments, the repeatability of single-probe qPCR was calculated, and the CV values were < 1.0% (0.04–0.90%) (Supplemental Table 3).To assess the detectability of this single-probe qPCR, we used DNA plasmids templates with five different ratios of *Wt* or *Mut* template to mixed template (100/100, 75/100, and 25/100 to 1/100) and determined that most of probes could distinguish as low as 1% *Wt* or *Mut* DNA from mixed templates of LHON mutation sites except the m.11778G>A *Wt* probe (Fig. 1F). These data suggest a strong detection ability of the single-probe qPCR system.

### Duplex TaqMan-MGB probe qPCR

**Figure 2:**
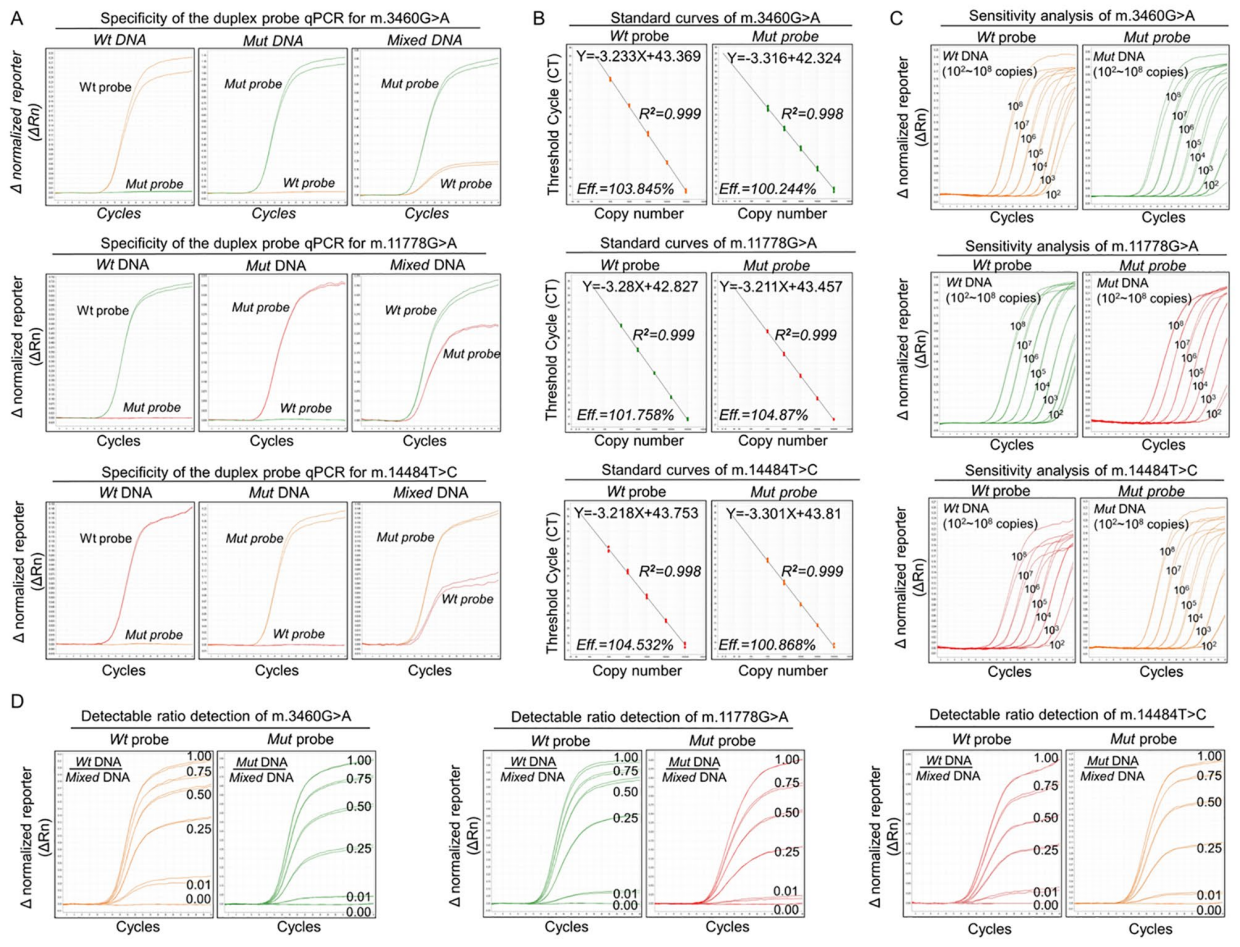
Duplex TaqMan-MGB Probe qPCR for Three LHON mtDNA Mutations. The duplex TaqMan-MGB probe qPCR was established using double probes (*Wt* and *Mut* probe) targeting the same LHON mtDNA mutation to simultaneously detect *Wt, Mut* or mixed DNA sequences of mtDNA mutations in three independent reactions. (**A**) Specificity analysis of the duplex-probe qPCR system. Duplex *Wt* or *Mut* probe were used to detect different DNA templates (*Wt, Mut* or mixed) of three mtDNA mutations. (**B**) Standard curves of the duplex-probe qPCR system. Five tenfold serial dilutions of *Wt* or *Mut* DNA templates (10^3^–10^7^ copies) in the duplex-probe system were used to construct the standard curves in three independent reaction tubes as described earlier. Representative amplification curves, Eff% and R^2^ are shown. (**C**) Sensitivity analysis of the duplex-probe qPCR system. The sensitivity of this duplex-probe qPCR was determined by detecting the DNA templates with a tenfold dilution limitation from 10^2^–10^8^ copies in three independent reactions with three replicates of each dilution used. (**D**) Detectable ratio analysis of the duplex-probe qPCR system. The detectable ratio of the duplex-probe qPCR was assessed using plasmid DNA templates with five different ratios of *Wt* or *Mut* templates to mixed templates (100/100, 75/100, and 25/100–1/100). All template quantities had 10^8^ copies and the experiments performed in triplicate. Representative curves are shown. *Wt* Wild type, *Mut* mutation, eff%, Efficiencies, *R*^2^ Coefficient of determination.

To determine whether duplex (*Wt* and *Mut*) probes of each mutation could simultaneously differentiate different mutation states in the same reaction, we then designed a duplex-probe qPCR in which *Wt* and *Mut* probes were combined to detect corresponding mtDNA mutation in the same PCR reaction. Specificity testing showed that the duplex-probe assay could specifically differentiate *Wt* and *Mut* DNA of each mtDNA mutation without cross-reactivity between *Wt* and *Mut* probes in the same reaction (Fig. 2A). Next, standard curves generated from the duplex-probe system also indicated excellent linear correlations between CT values and DNA loading copy numbers with similar R^2^ and Eff% from a single-probe assay (Fig. 2B). Furthermore, sensitivity testing showed that the duplex-probe qPCR could simultaneously detect DNA templates as low as 10^2^ copies in the same PCR reaction (Fig. 2C). Based on the results obtained from each dilution of standard plasmid DNA in five different experiments, the repeatability of qPCR was calculated and the CV values were < 1.0%, ranging from 0.29% to 0.99% (Supplemental Table 4). Moreover, detectable ratio analysis indicated that the duplex-probe system also could detect as low as 1% *Wt* or *Mut* DNA from mixed templates, which is similar to the single-probe qPCR system (Fig. 2D). Together, these data suggest that the duplex-probe qPCR possesses high specificity, sensitivity and repeatability without cross-reactivity between *Wt* and *Mut* probes.

### Triplex TaqMan-MGB probe qPCR

**Figure 3:**
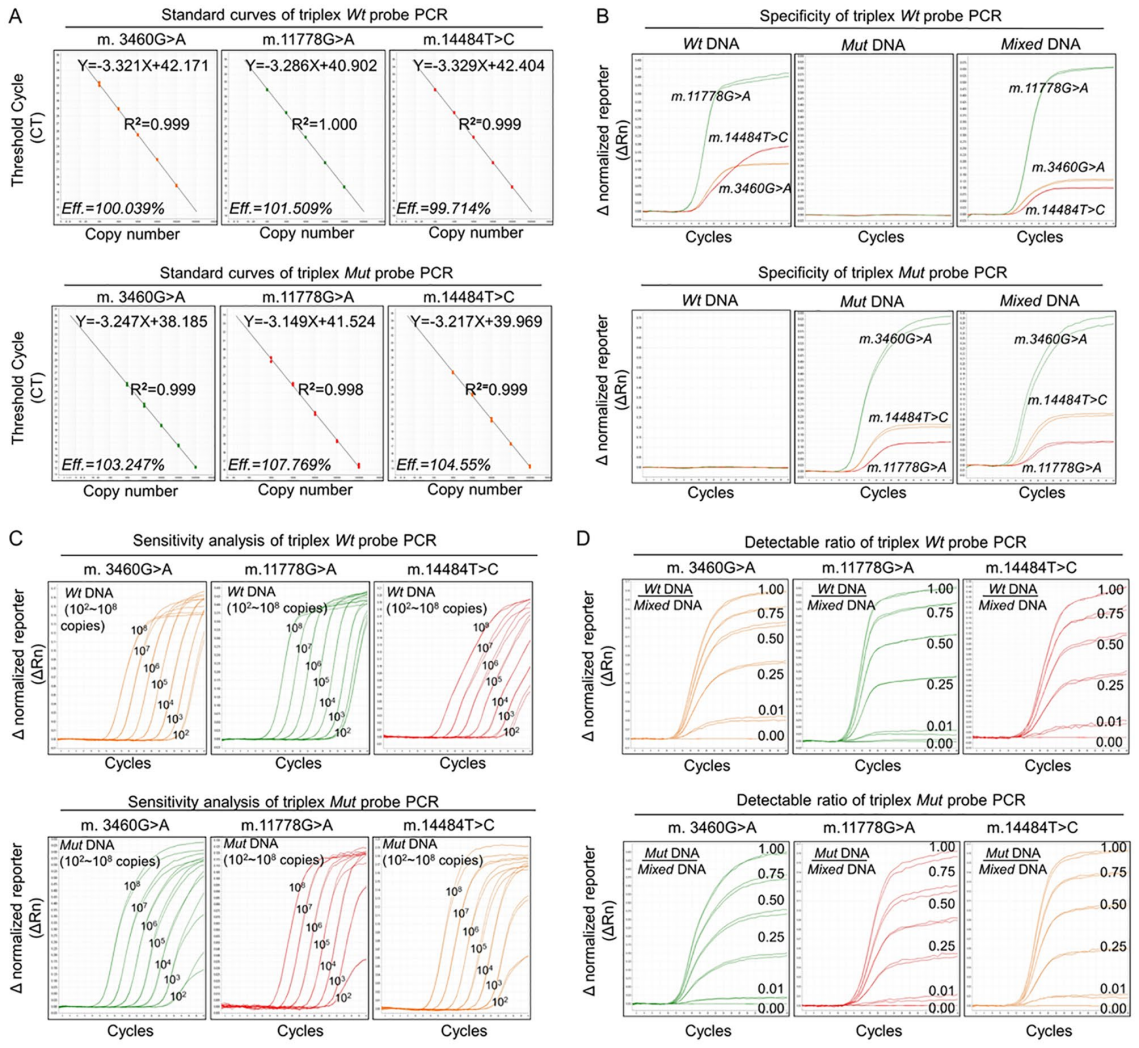
Triplex TaqMan-MGB probe qPCR for Three LHON mtDNA Mutations. The triplex TaqMan-MGB probe qPCR was established using triplex *Wt* or *Mut* probe targeting three LHON mtDNA mutations to simultaneously detect *Wt, Mut* or mixed DNA of three mtDNA mutations in two independent PCR reactions. (**A**) Standard curves of the triplex-probe qPCR system. Five tenfold serial dilutions (10^3^–10^7^ copies) of three mixed DNA templates composed of *Wt* or *Mut* plasmids of three mutations were amplified with the triplex *Wt* or *Mut* probe system in two independent PCR reactions. (**B**) Specificity analysis of the triplex-probe qPCR system. Triplex *Wt* or *Mut* probe was used to detect different templates (*Wt*, *Mut* or mixed DNA) of three mtDNA mutations. (**C**) Sensitivity analysis of the triplex-probe qPCR system. The sensitivity of triplex-probe qPCR was determined by detecting three LHON mtDNA mutations with a tenfold DNA dilution limitation from 10^2^ to 10^8^ copies in two independent PCR reactions with three *Wt* or *Mut* probes. Three replicates of each dilution were used. (**D**) Detectable ratio analysis of the triplex-probe qPCR system. The detectable ratio of the triplex-probe qPCR was assessed using plasmid DNA templates with five different ratios of *Wt* or *Mut* DNA to mixed DNA (100/100, 75/100, and 25/100–1/100). All template quantities were in 10^8^ copies and were performed in triplicate. Representative curves are shown. *Wt* Wild type, *Mut* mutation, *Eff%* Efficiencies, *R*^2^ coefficient of determination.

To further reduce reaction tubes through cost-saving advantage, we finally set up a triplex*-*probe qPCR system for three mtDNA mutations in two PCR reactions, in which the combination of triplex *Wt* or *Mut* probe was employed to simultaneously distinguish three different *Wt* or *Mut* DNA templates. Meanwhile, standard curves generated from the same PCR reaction all showed excellent linear correlations between CT values and the DNA loading copy number (*Wt* probe group: R^2^ = 0.999–1.000; Eff% = 99.714–101.519%; *Mut* probe group: R^2^ = 0.998–0.999; Eff% = 103.247–107.769%) (Fig. 3A), which are similar with those from single- or duplex-probe qPCR systems. We also further found that standard curves either from genomic DNA or plasmid DNA presented very close R^2^ and Eff% values (Supplemental Fig. 4A–4B), indicating that the triplex-probe TaqMan qPCR system features a better capacity of resisting disturbance of genomic DNA. Specificity analysis further showed that the triplex *Wt* or *Mut* probes could specifically differentiate corresponding *Wt* or *Mut* templates of three mtDNA mutations without cross-interferences (Fig. 3B). We also found that the triplex-probe qPCR could simultaneously detect DNA templates as low as 10^2^ copies in the same reaction with mixed DNA templates (Fig. 3C) while also detect as low as 1% *Wt* or *Mut* DNA from mixed DNA templates (Fig. 3D), indicating a better sensitivity and detectable ability. Based on the results obtained from each dilution of standard plasmid DNA in five different experiments, the repeatability was calculated and the CV values were < 1.0%, ranging from 0.03 to 0.92% (Supplemental Table 5). Overall, these results suggest that the triplex-probe qPCR assay presents excellent specificity, sensitivity and reproducibility without cross-reactivity between the *Wt* or *Mut* probes.

### Validation of the TaqMan-MGB probe qPCR assay on clinical samples

As the triplex-probe TaqMan qPCR assay presented excellent amplification efficiency with time and cost-saving advantages, we further considered validating this triplex-probe assay using clinical blood samples. As the copy number ratio of the 12 s rRNA gene to the β-globin gene is widely used for measuring relative copy number ratios of mtDNA to genomic DNA in blood samples^7,19^, we further generated two standard curves for 12 s rRNA and β-globin gene using SYBR Green qPCR method (Supplemental Fig. 5A). According to these standard curves, we analyzed the mtDNA copy number as well as that for genomic DNA and obtained the relative copy number ratio of mtDNA to genomic DNA in blood samples (Supplemental Fig. 5C). After eliminating outliers from experimental errors, we finally determined the mean value of mtDNA copy number at 84,008 ± 29,543 copies/5 ng total DNA (Supplemental Table 6). Furthermore, we found that these mtDNA copy numbers from different blood samples followed a typical Gaussian distribution (Supplemental Fig. 5B) and the mean copy number ratio of 12 s rRNA/β-globin is 5.8 ± 2.11 (Supplemental Fig. 5C).

**Figure 4:**
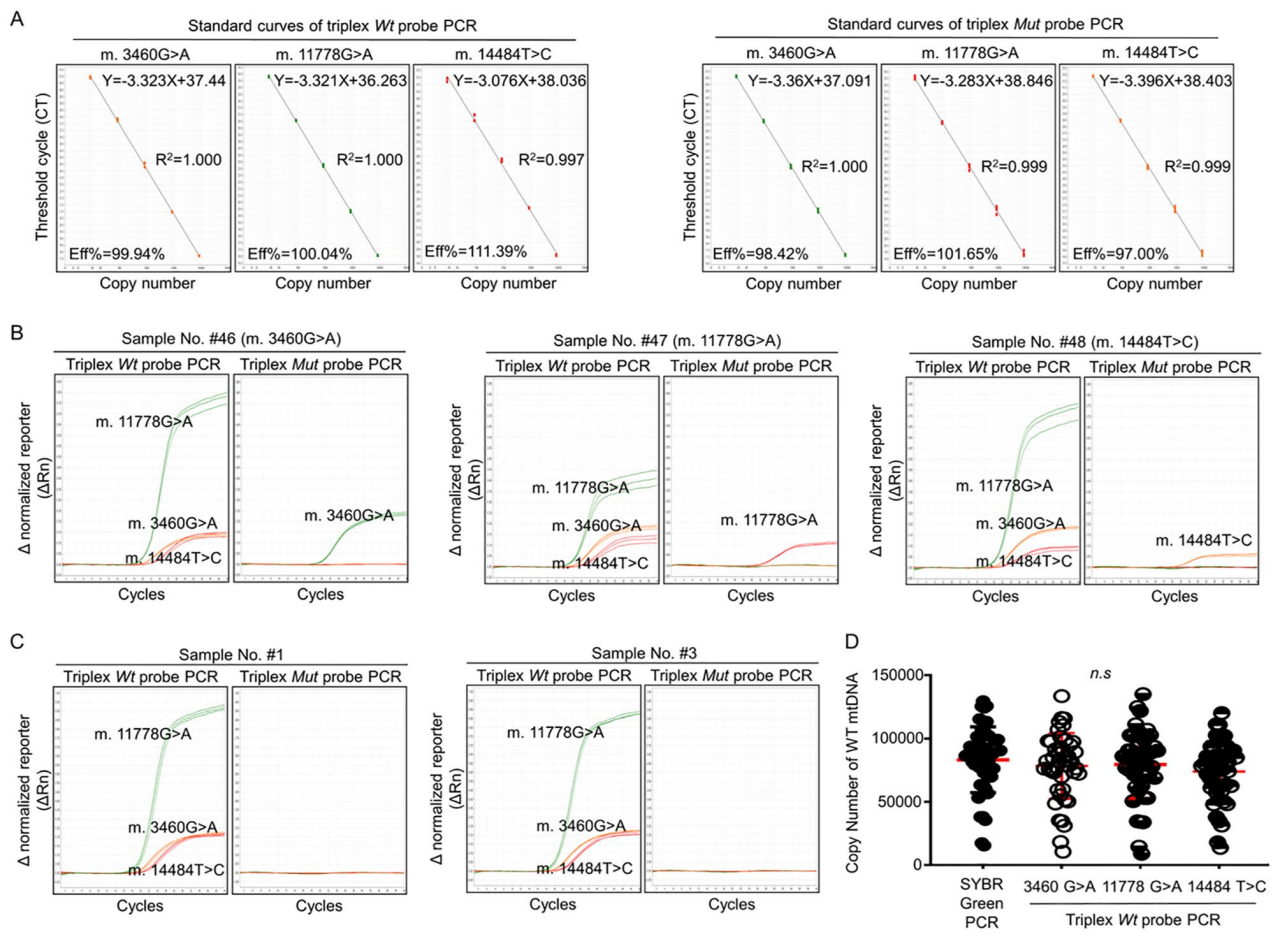
Validation of the Triplex-probe qPCR System for LHON mtDNA Mutations Using Clinical Samples. Total DNA from 48 clinical blood samples was amplified with the triplex-probe qPCR system. Standard curves from the same PCR reaction plate were constructed using five tenfold serial dilutions (10^3^–10^7^copies) of *Wt* or *Mut* standard plasmid templates (**A**). Representative amplification curves from three LHON mtDNA mutations (Sample No. #46, #47 and #48) (**B**) and unaffected individuals (Sample No. #1 & #3) (**C**) are shown. The quantitative ability of triplex-probe qPCR assay for mtDNA copy number was compared with SYBR Green qPCR using dual-blind, grouped clinical DNA samples (**D**). Representative curves are shown. *Wt* Wild type, *Mut* mutation, *Eff%* Efficiencies, *R*^2^ coefficient of determination, *n.s.* no significant.

Based on these data, we further established the standard curves of the triplex-probe TaqMan qPCR system (Fig. 4A) and validated the triplex-probe qPCR assay by qualitative and quantitative testing. Using dual-blind grouped samples, we found that the triplex-probe TaqMan qPCR could accurately genotype three mutation carriers as well as unaffected individuals (Fig. 4B, C and Supplemental Table 6). Meanwhile, by comparing the quantitative results from blood samples, we found that the mtDNA copy number in these samples had no significant difference between the triplex-probe qPCR and SYBR Green qPCR (Fig. 4D and Supplemental Table 7). To further verify whether this triplex-probe TaqMan qPCR could distinguish the different content of mtDNA mutations, we set up 16 samples with three levels of mutation contents by mixing the *Mut* plasmid DNA and genomic DNA in the different proportions (three samples of 0–25%, 10 samples of 25–80% and three samples of 80–95%). As shown in the Supplemental Table 8, the triplex-probe TaqMan qPCR presented an enhanced ability to distinguish different *Mut* mtDNA levels. Together, these data indicate the feasibility of the triplex-probe TaqMan qPCR for analysis of clinical samples.

## Discussion

Developing methods of genetic diagnosis with low-cost, less turnover time, and reliable single nucleotide polymorphism (SNP) determination, has been attracting increasing interest in the age of personalized medicine. It is widely acknowledged that using information from multiple SNP genotypes provides a more accurate risk assessment than that predicted by a single risk allele^23^. The incomplete penetrance of LHON with heteroplasmic mutations causes considerable undetected asymptomatic carriers with LHON mutations in the general populations, increasing their risk of losing vision. Thus, a unique consideration for mtDNA mutations analysis should be reliability and sensitivity of qualitative genotyping and quantitative detection for low levels of heteroplasmic mutations from clinical samples^8^*.*

T-ARMS-PCR is identified as one of the most common methods for SNP genotyping heteroplasmic and homoplasmic mutations without additional post-PCR manipulation^20,21^. In this study, a “one-step” T-ARMS-PCR assay was developed to identify three LHON mtDNA mutations rapidly and qualitatively, with better specificity, sensitivity, and amplification efficiency within less than three hours and with minimal operation. This T-ARMS-PCR assay is a fast, economical, and reproducible alternative to other expensive assays for the qualitative detection of the LHON mtDNA mutations. Thus, it might be suitable for conventional laboratories that cannot afford expensive and sophisticated equipment as well as high-cost technical expertise. However, this assay is only used for qualitative genotyping rather than quantitative detection. Thus, the availability of a reliable quantitative assay for heteroplasmic evaluation is particularly critical to screen its somatic and germline segregation of the LHON mtDNA mutations in patients and their families.

Fluorescence-labelled MGB probe qPCR has been recently developed to quantify mtDNA molecules with greater specificity and sensitivity than conventional DNA probes, especially for single-base mismatches that occur in the MGB region of the duplex^24,25^. Our study further established a series of TaqMan-MGB probe qPCR assays to perform both qualitative genotyping and quantitative detection of three LHON mtDNA mutations, serving as a highly promising approach for fast, cost-saving and accurate diagnosis of LHON. The combination of primers and probes plays a crucial role in the amplification of the TaqMan MGB probe qPCR, especially for the multiplex qPCR system. Therefore, primers and probes in our study required more comprehensive optimization, specifically for multiplex probe qPCR assays. Firstly, the Tm value of MGB probes is designed to be 8 °C higher than the primers, making the probes stably bind to the templates prior to the primers. Moreover, all MGB probes were designed to be shorter than conventional probes with similar Tm values as a single base site mutation may cause a huge change in Tm, resulting in the unstable binding of the MGB probes to the templates. Further, the GC contents of the primers and probes were optimized between 40 and 60% so that the target DNA can be efficiently amplified. Meanwhile, probes were labelled with FAM, NED, or CY5 fluorophores to prevent the detection signals from overlapping in the multiplex-probe qPCR assay, thereby avoiding non-specific fluorescent signals for qPCR assay and the use of radioactively labelled nucleotides for hybridation assay. Based on these optimizations, our results suggest that the integration of two or three labelled probes in an equimolar ratio does not significantly compromise the basic parameters of duplex- or triplex-probe qPCR assays corresponding to single-probe counterparts, thus, presenting a better amplification ability with an identical specificity and stable repeatability without cross-reactivity. More notably, whatever single-probe or multiplex-probe combinations, these TaqMan MGB-probe qPCR assays showed both stable amplification efficiency and sensitivity in different reaction systems. Furthermore, three qPCR assays can detect as low as 1% of *Wt* or *Mut* DNA in mixed standard plasmid templates, demonstrating an ability to trace low copy number mutations in pedigrees. Previous studies have reported the SYBR Green and TaqMan probe-based ARMS real-time PCR for LHON mutation^25–27^. However, these approaches were used to only define m.3243A>G site rather than three primary LHON mutations. Moreover, a recent report also described a TaqMan MGB probe qPCR only for m.3460G>A mutation^28^, limiting its application for the detection of three major LHON mtDNA mutations. Our data from the present study suggest that the TaqMan-MGB probe qPCR assay has excellent specificity, sensitivity, and repeatability with stable amplification efficiency with respect to the corresponding single-probe counterparts.

The major advantages of the triplex-probe approach do not only lie in the improvement of the speed and cost-savings of the application itself, but also reliable assessment as a first-line screening tool for clinical samples. Thus, we focused our efforts to establish a triplex-probe qPCR assay for three LHON mtDNA mutations in clinical blood samples. The feasibility of the TaqMan PCR assay was validated in 48 clinical samples using conventional SYBR Green PCR and the TaqMan probe assay. By analyzing the ratio of the mitochondrial 12 s rRNA to genomic β-globin in total DNA, the mtDNA copy number from blood samples was determined using the SYBR Green qPCR assay as previously reported. Subsequently, double-blind, grouped DNA samples were subject to further triplex-probe qPCR testing. Considering genomic DNA in total DNA may exert non-specific interference with a qPCR assay, so we compared the amplification efficiency of triplex-probe qPCR using plasmid and genomic DNA as templates. However, we did not find significant differences between them. To verify its ability to detect *Mut* mtDNA, we specifically set up three levels content of mtDNA mutations in total DNA and also confirmed the robust ability of this qPCR to detect different mutation contents from heteroplasmic DNA. Thus, these data suggest that the triplex-probe assay can accurately distinguish the varying content of mtDNA mutations without regard to the presence of genomic DNA.

However, there are some additional limitations in our study. First, a small sample size with LHON mutations limited a comprehensive evaluation of this qPCR in this study. Therefore, expanding sample sizes is required to perform a more thorough evaluation and validation in the next pre-clinical testing. Then, other methods should be developed to compare the measurement of the *Mut* mtDNA copy number from the TaqMan probe qPCR. In addition, it is important to highlight that these probe-based qPCR assays cannot exclude other mutations except three primary LHON mtDNA mutations. Extensive research concerning other high frequent mtDNA mutations remains to be conducted, in addition to in-depth studies of the association between these mutations and clinical phenotypes, to obtain a better understanding of the underlying molecular pathology, and guide the development of new strategies to minimize the effects of the mutations.

In summary, our data describes a qualitative T-ARMS-PCR assay to define mutation states of three primary LHON mtDNA mutations, allowing a routine pre-screening analysis of three primary LHON mutations in conventional laboratories with limited resources. Moreover, our study demonstrates a specific, sensitive, robust and reproducible TaqMan-MGB probe qPCR assay for simultaneous qualitative and quantitative analysis of three major LHON mutations, offering a rapid, low cost, easy to handle, and high-throughput approach with potential applications in both clinical as well as academic research settings for genetic screening and testing of LHON mutations.

## Supplementary information

Supplementary information.
