# Supplementary material for: TaqMan-MGB probe quantitative PCR assays to genotype and quantify three mtDNA mutations of Leber hereditary optic neuropathy
Source: Sci Rep. 2020 Jul 23;10:12264. doi: 10.1038/s41598-020-69220-7 (PMC7378831; doi:10.1038/s41598-020-69220-7)

TaqMan-MGB probe quantitative PCR assays to genotype and quantify three mtDNA mutations of Leber Hereditary Optic Neuropathy

Bingqian Xue [1], Yang Li [1,2], Xin Wang[1], Rui Li [1], Xin Zeng[1], Meihua Yang[3], Xiaohui Xu[1], Tingbo Ye [1,4], Liming Bao[5], Yi Huang [1] ✉

[1] Chongqing Key Laboratory of Child Infection and Immunity, Ministry of Education Key Laboratory of Child Development and Disorders, National Clinical Research Center for Child Health and Disorders, China International Science and Technology cooperation base of Child development and Critical Disorders, Children's Hospital of Chongqing Medical University, Chongqing 400014, China; [2] Department of Laboratory Medicine, Zhengzhou Central Hospital Affiliated to Zhengzhou university，Zhengzhou 450007，China; [3] Department of Neurosurgery, Xinqiao Hospital of Army Military Medical University, Chongqing 400037, China; [4] The Third People's Hospital of Chengdu, Chengdu 610031, China; [5] Department of Pathology, University of Colorado School of Medicine, Aurora, CO 80045, USA

✉ **Correspondence**: Yi Huang, Children's Hospital of Chongqing Medical University; No.136 Zhongshan Erd Road, Yuzhong District, Chongqing 400014, China; Phone: +86-23 -63612114; Fax: +86-23-63622754; Email: yihuang828@foxmail.com or yihuang@hospital.cqmu.edu.cn;

Supplemental Figures and Figure legends:

Figure.S1

Figure.S2

Figure.S3

Figure.S4

Figure.S5

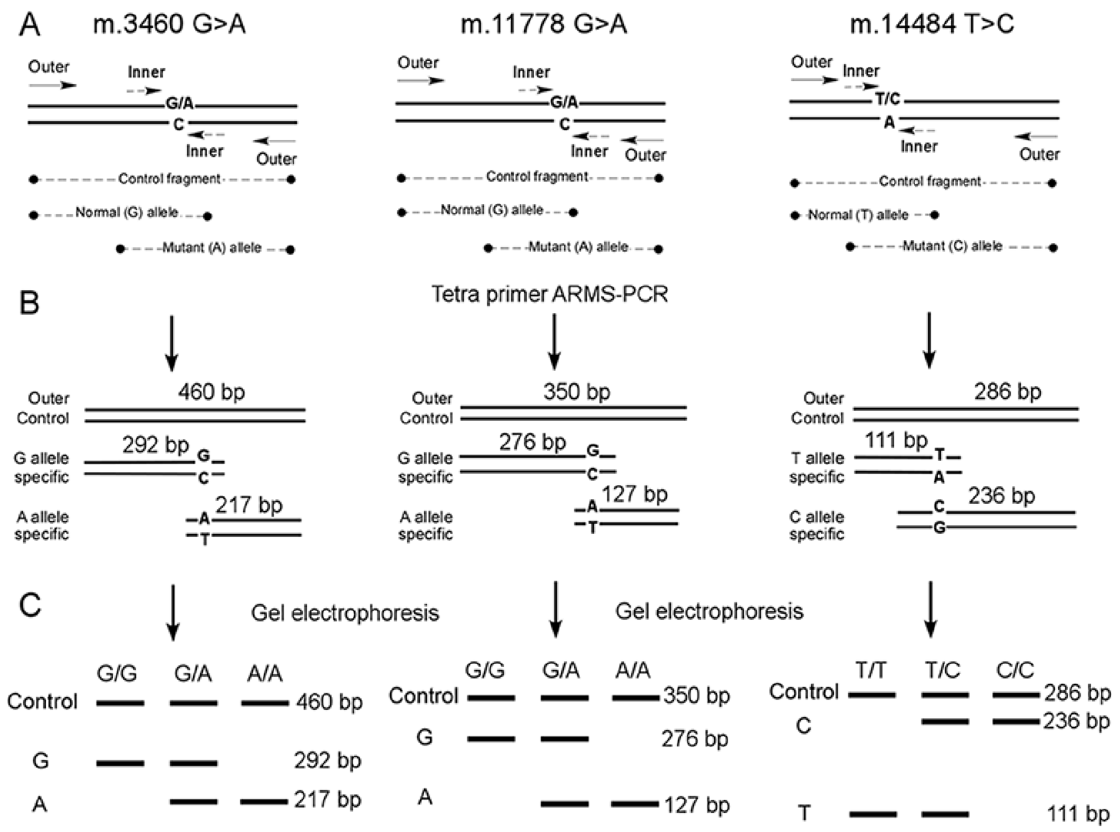

Figure.S1 Schematic Diagrams of T-ARMS PCR for Three LHON mtDNA Mutations.

(A) A larger (non-allele-specific) control fragment and two different allele-specific amplicons are generated by a pair of two outer primers and two inner (allele-specific) primers that have opposite orientation. (B) By positioning the two outer primers at different distances in mtDNA from the polymorphic nucleotide, the two allele-specific amplicons differ in length by PCR. (C) The expected positions of the DNA control, mutant, and normal alleles are indicated after gel electrophoresis. The fragments are not drawn to scale.

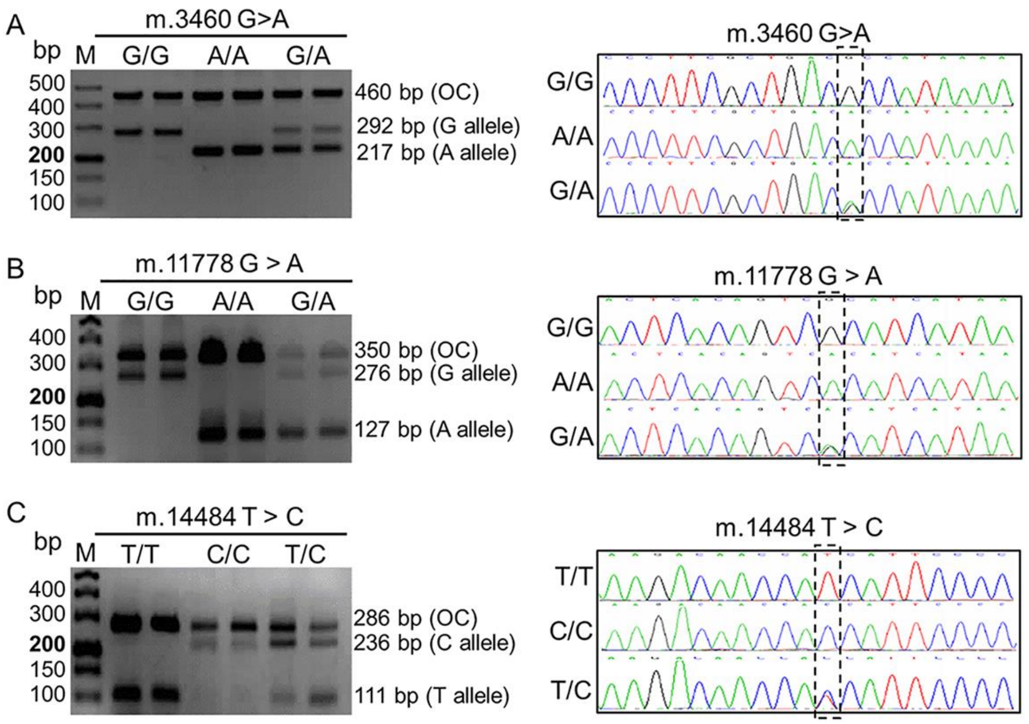

Figure.S2 Optimized T-ARMS-PCR for the Qualitative Detection of Three LHON mtDNA Mutations.

T-ARMS-PCR was optimized to detect the three LHON mtDNA mutations using standard plasmid DNA. All the mutations were further confirmed by direct DNA sequencing. PCR products were separated on 2.5% agarose gel. Representative gel electropherogram of PCR products and DNA sequencing results for mutations m.3460G>A (A), m.11778G>A (B) and m.14484T>C (C) are shown respectively. M: DNA loading markers. The black framework indicates the mutants' position.

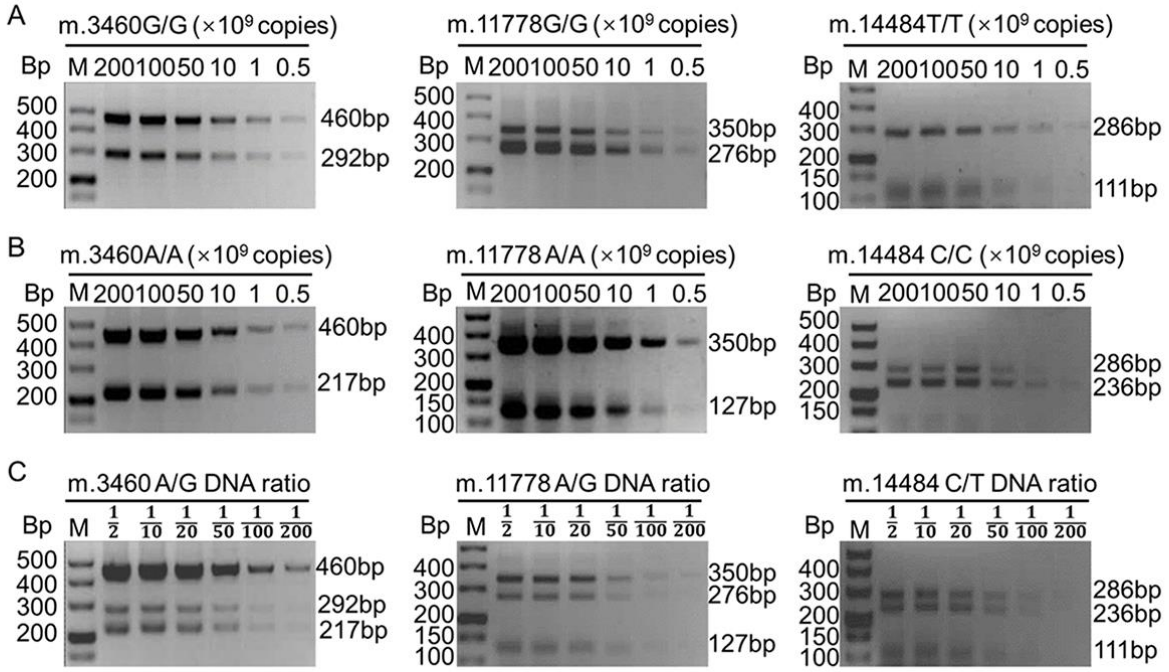

Figure.S3 Sensitivity of T-ARMS-PCR for Three LHON mtDNA Mutations.

Standard plasmid DNA were used to reach different DNA amount per 25 μl PCR volume, with a stepwise decrease from 200×10^9 to 0.5×10^9 copies plasmid DNA, to determine the smallest detectable amount of *Wt* DNA (m.3460G/G, m.11778G/G, and m.14484T/T) (A) and *Mt* DNA (m.3460A/A; m.11778A/A and m.14484C/C) (B) by optimized T-ARMS-PCR assay. To analyze sensitivity of T-ARMS-PCR assay for the detection of heteroplasmic LHON mutations, we mix genomic DNA containing known amounts of *Wt* mtDNA with *Mut* standard plasmid and the ratios of *Wt to Mut* DNA *were* from 1:2 – 1:200. (C). All PCR products were separated on 2.5% agarose gel. M: DNA markers.

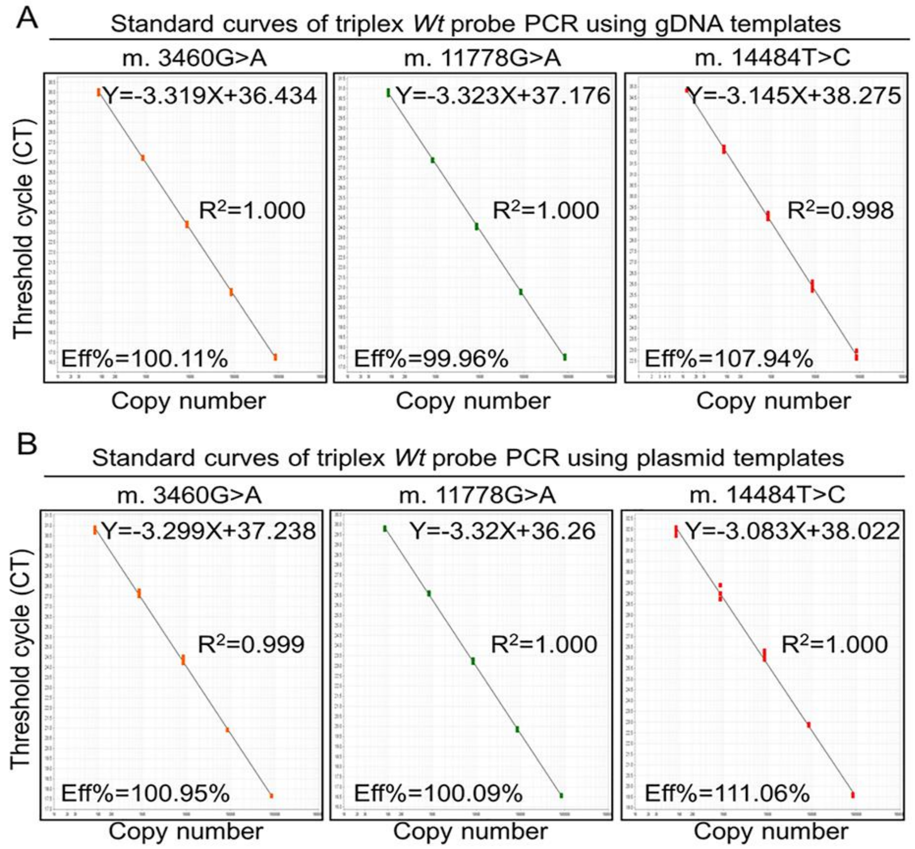

Figure. S4 Comparison of Amplification Efficiency of the Triplex-probe qPCR System Between Plasmid DNA and Genomic DNA Templates.

The amplification efficiency of the triplex-probe qPCR system was compared between plasmid DNA and genomic DNA (gDNA) templates. Five 10-fold serial dilutions (5×10^{-4} –5×10^{0} ng) of gDNA (A) and five 10-fold serial dilutions (8.4×10^{1}–8.4×10^{5} copies) of standard *Wt* plasmids (B) were amplified with triplex *Wt* probe system respectively. Representative standard curves are shown. *Wt*, Wild type; *Mut*, mutation; Eff%, Efficiencies; R^2, Coefficient of determination.

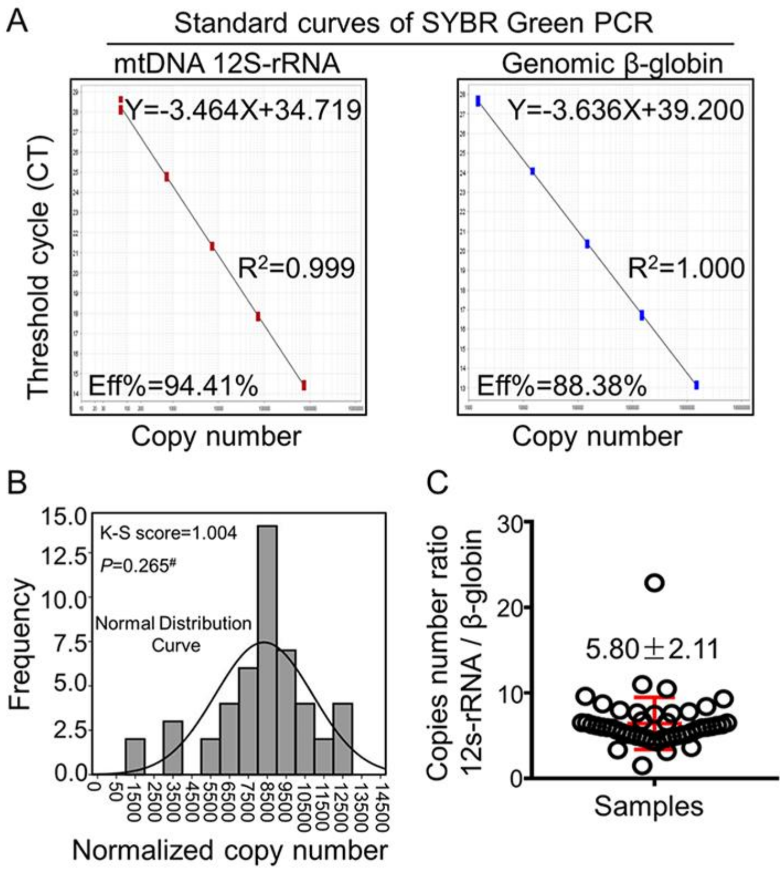

Figure.S5 Determination of mtDNA Copy Number by SYBR Green qPCR.

SYBR Green qPCR was used to determine copy number of mtDNA in 5.0 ng total DNA from blood samples by analyzing the 12s rRNA gene. β-globin gene was used for genomic DNA (gDNA) control. Five 10-fold serial dilutions from purified PCR products of 12s rRNA (7.34×10^1–7.34×10^5 copies) and β-globin (1.5×10^3–1.5×10^7 copies) were used to generate DNA standard curves (A). The mtDNA copy number detected by SYBR Green qPCR was subject to Gaussian distribution analysis (B) and the mtDNA copy number relative to to gDNA was calculated (C). Representative curves are shown. *Wt*, Wild type; *Mut*, mutation; Eff%, Efficiencies; R^2, Coefficient of determination; $^{\#}$ P>0.05 indicate that the distribution curve obeys a Gaussian distribution.

Supplemental Tables

Supplemental Table 1
Supplemental Table 2
Supplemental Table 3
Supplemental Table 4
Supplemental Table 5
Supplemental Table 6
Supplemental Table 7
Supplemental Table 8

Supplemental Table 1. Primers of SYBR Green qPCR

Gene (Gene ID)	Primer Sequence (5′-3′)	Tm (℃)	Annealing Tm (℃)	Amplicon Size (bp)
12s rRNA (ID: 6775087)	F: ACAATTCTCCGATCCGTCCC	59.5	60	136
	R: GTGATTGGCTTAGTGGGCGA	60.4		
β-globin (ID: 3403)	F: GCTCGGTGCCTTTAGTGATG	59.0	60	134
	R:ATCAAGCGTCCCATAGACTCA	58.6		

F, forward; R, reverse; Tm: Temperature;

Supplemental Table 2. Reaction components of TaqMan-MGB probe qPCR system

Genetic Polymorphism	Primers & Probes	Single probe qPCR (μM)						Duplex probe qPCR (μM)			Triplex probe qPCR (μM)	
		m. 3460G>A *Wt*	m.11778G>A *Wt*	m.14484T>C *Wt*	m. 3460G>A *Mut*	m.11778G>A *Mut*	m.14484T>C *Mut*	m. 3460G>A	m.11778G>A	m.14484T>C	*Wt* mtDNA	*Mut* mtDNA
m. 3460G>A	F-primer	0.20			0.20			0.20			0.10	0.10
	R-primer	0.20	/	/	0.20	/	/	0.20	/	/	0.10	0.10
	Wt probe	0.05			/			0.05			0.05	/
	Mut probe	/			0.05			0.05			/	0.05
m.11778G>A	F-primer		0.20			0.40			0.40		0.10	0.20
	R-primer		0.20			0.40			0.40		0.10	0.20
	Wt probe	/	0.05	/	/	/	/	/	0.05	/	0.05	/
	Mut probe		/			0.20			0.20		/	0.10
m.14484T>C	F-primer			0.40			0.20			0.40	0.20	0.10
	R-primer	/	/	0.40	/	/	0.20	/	/	0.40	0.20	0.10
	Wt probe			0.20			/			0.2	0.10	/
	Mut probe			/			0.05			0.05	/	0.05

Note: MT: mitochondrial; *Mut*: mutation; *Wt*: Wild type; ND: NADH dehydrogenase; F: Forward; R: Reverse; μM: Final concentration;

Supplemental Table 3. Repeatability of the single probe qPCR for LHON mtDNA mutations

Dilution (Copies)	m. 3460G>A *Wt*		m.11778G>A *Wt*		m.14484T>C *Wt*		m. 3460G>A *Mut*		m.11778G>A *Mut*		m.14484T>C *Mut*	
	CT ± SD	CV%	CT ± SD	CV%	CT ± SD	CV%	CT ± SD	CV%	CT ± SD	CV%	CT ± SD	CV%
10^7	19.83±0.06	0.29	18.15±0.02	0.10	21.49±0.06	0.27	17.70±0.06	0.32	19.93±0.16	0.83	19.88±0.90	0.90
10^6	23.21±0.09	0.37	21.49±0.03	0.15	24.79±0.04	0.16	20.91±0.03	0.14	23.31±0.06	0.24	23.17±0.41	0.41
10^5	26.64±0.11	0.43	24.95±0.01	0.04	28.07±0.21	0.76	24.03±0.07	0.30	26.65±0.10	0.36	26.27±0.59	0.59
10^4	29.87±0.14	0.46	28.28±0.11	0.39	31.47±0.03	0.10	27.37±0.06	0.21	29.75±0.13	0.43	29.52±0.28	0.28
10^3	33.42±0.15	0.46	31.14±0.17	0.54	34.01±0.22	0.63	30.49±0.14	0.45	33.21±0.17	0.51	32.83±0.07	0.21

Note: CV, coefficients of variance; SD, standard deviation; *Wt*, wild type DNA; *Mut*, mutation DNA;

Supplemental Table 4. Repeatability of the duplex-probe qPCR for LHON mtDNA mutations

Dilution (Copies)	m.3460G>A *Wt*		m.3460G>A *Mut*		m.11778G>A *Wt*		m.11778G>A *Mut*		m.14484T>C *Wt*		m.14484T>C *Mut*	
	CT ± SD	CV%	CT ± SD	CV%	CT ± SD	CV%	CT ± SD	CV%	CT ± SD	CV%	CT ± SD	CV%
10^7	20.72±0.12	0.56	19.15±0.17	0.89	19.77±0.08	0.39	20.67±0.10	0.48	21.12±0.15	0.72	20.90±0.11	0.53
10^6	24.06±0.19	0.78	22.45±0.18	0.80	23.12±0.07	0.29	23.90±0.15	0.62	24.43±0.24	0.96	24.20±0.14	0.57
10^5	27.33±0.10	0.35	25.77±0.26	0.99	26.37±0.15	0.57	27.28±0.26	0.96	27.71±0.22	0.78	27.39±0.17	0.62
10^4	30.32±0.16	0.53	29.02±0.26	0.90	29.79±0.09	0.31	30.68±0.21	0.67	30.72±0.15	0.49	30.64±0.11	0.37
10^3	32.25±0.17	0.52	32.31±0.27	0.83	32.87±0.21	0.64	34.03±0.14	0.43	33.88±0.18	0.52	33.80±0.12	0.37

Note: CV, coefficients of variance; SD, standard deviation; *Wt*, wild type DNA; *Mut*, mutation DNA;

Supplemental Table 5. Repeatability of the triplex-probe qPCR for LHON mtDNA mutations

Dilutions (Copies)	m. 3460G>A *Wt*		m.11778G> A *Wt*		m.14484T>C *Wt*		m. 3460G>A *Mut*		m.11778G>A *Mut*		m.14484T>C *Mut*	
	CT ± SD	CV%	CT ± SD	CV%	CT ± SD	CV%	CT ± SD	CV%	CT ± SD	CV%	CT ± SD	CV%
10^7	18.89±0.05	0.27	17.86±0.03	0.15	19.14±0.06	0.31	15.56±0.08	0.52	19.35±0.05	0.28	17.34±0.05	0.27
10^6	22.29±0.02	0.09	21.14±0.02	0.09	22.38±0.06	0.27	18.68±0.06	0.33	22.54±0.12	0.53	20.65±0.11	0.53
10^5	25.54±0.04	0.17	24.55±0.01	0.06	25.79±0.03	0.10	21.93±0.20	0.92	25.94±0.10	0.37	24.02±0.10	0.43
10^4	28.96±0.05	0.17	27.85±0.01	0.03	29.05±0.17	0.60	25.20±0.16	0.63	28.89±0.25	0.87	27.03±0.08	0.29
10^3	32.15±0.23	0.72	30.94±0.03	0.10	32.44±0.21	0.65	28.53±0.07	0.23	31.97±0.11	0.34	30.32±0.09	0.30

Note: CV, coefficients of variance; SD, standard deviation; *Wt*, wild type DNA; *Mut*, mutation DNA;

Supplemental Table 6. The copy number of mtDNA in 48 blood samples by SYBR Green qPCR

Sample ID#	Gender	CT ± SD (12s rRNA)	CT ± SD (β-globin)	CN ± SD (12s rRNA)	CN ± SD (β-globin)	Triplex probe qPCR genotyping
#01	M	17.23±0.40	23.82±0.32	114144.41±27569.65	17181.58±3551.85	None
#02	M	17.43±0.13	23.90±0.28	97991.94±8451.36	16362.60±3052.79	None
#03	F	18.34±0.22	25.42±0.42	54027.41±7468.23	6287.14±1575.48	None
#04	M	17.41±0.11	23.90±0.31	99756.42±7069.95	16351.89±3125.05	None
#05	M	18.22±0.42	23.71±0.43	59466.5±16645.33	18675.72±4626.20	None
#06	M	17.60±0.32	23.81±0.25	89009.37±17574.10	17220.43±2587.35	None
#07	F	17.53±0.43	24.02±0.41	93908.82±24967.06	15356.89±4156.66	None
#08	M	17.30±0.14	24.16±0.17	106865.2±10007.49	13720.90±1561.71	None
#09	M	17.33±0.17	23.77±0.04	105280.37±11607.17	17516.31±387.63	None
#10	M	17.06±0.29	23.69±0.36	126680.16±23070.80	18800.90±4091.59	None
#11	F	17.52±0.28	24.03±0.11	93547.58±16993.21	14895.53±1011.69	None
#12	M	17.88±0.44	23.44±0.45	74957.40±22466.35	22208.31±5858.25	None
#13	M	17.19±0.42	23.39±0.43	118044.96±30351.15	22876.26±5840.88	None
#14	M	17.38±0.40	23.76±0.14	103550.24±25612.31	17670.20±1638.68	None
#15	M	17.48±0.33	23.45±0.38	96290.06±20424.81	21877.19±5496.14	None
#16	M	17.09±0.41	23.60±0.44	125572.4±31498.19	20046.24±5641.56	None
#17	M	17.52±0.25	23.44±0.45	92984.31±15773.25	22237.63±6054.39	None
#18	F	17.52±0.37	23.80±0.43	94542.34±24821.61	17618.90±4650.33	None
#19	M	18.01±0.29	24.2±0.24	67321.52±12361.79	13422.92±2004.63	None
#20	F	17.90±0.43	23.99±0.36	73602.06±21144.71	15497.82±3406.18	None
#21	F	19.25±0.41	26.74±0.43	29999.55±7977.18	2731.30±750.63	None
#22	F	17.96±0.41	25.32±0.42	70499.13±18926.29	6726.51±1885.21	None
#23	M	17.59±0.26	24.04±0.06	88703.32±15785.21	14799.10±573.62	None
#24	M	17.01±0.29	23.57±0.45	130816.91±24168.93	20409.95±5720.87	None
#25	M	16.13±0.18	24.63±0.37	233261.12±28065.98	10329.40±2270.77	None
#26	F	17.67±0.21	24.03±0.07	84149.02±11730.59	14845.94±612.78	None
#27	M	17.51±0.43	24.10±0.40	95375.16±24901.78	14558.38±3732.32	None
#28	F	17.61±0.41	23.92±0.45	89152.53±22132.93	16374.57±4592.04	None
#29	M	17.94±0.14	24.01±0.10	69983.96±6874.89	15100.93±966.00	None
#30	M	17.81±0.34	24.03±0.27	77683.63±18623.48	15041.26±2417.16	None
#31	M	17.60±0.35	24.59±0.13	89177.32±20247.19	10433.95±833.02	None
#32	F	18.16±0.21	24.29±0.26	60790.75±8477.15	12709.27±2194.11	None
#33	M	17.91±0.13	24.08±0.32	71475.99±6271.67	14632.16±3137.81	None
#34	M	17.95±0.43	24.20±0.49	71053.45±18910.44	13816.47±4274.17	None
#35	M	17.51±0.37	23.95±0.39	95013.35±23112.43	15916.20±3655.95	None
#36	M	19.62±0.25	23.99±0.46	23047.01±3755.32	15676.79±4555.10	None
#37	M	20.31±0.33	26.56±0.46	14702.24±3360.40	3076.76±890.42	None
#38	M	17.26±0.42	24.16±0.48	112153.52±29254.33	14122.42±4257.57	None
#39	M	17.59±0.26	24.79±0.43	89039.56±15241.54	9381.71±2334.29	None
#40	M	17.97±0.19	24.52±0.26	69017.51±8682.97	11027.54±1708.93	None
#41	M	17.77±0.29	24.36±0.22	79051.18±14535.74	12132.54±1653.71	None
#42	F	18.06±0.32	24.52±0.24	65333.69±13409.39	10955.76±1729.29	None
#43	M	18.14±0.45	25.32±0.45	63002.54±18172.09	6746.99±1915.01	None
#44	M	17.87±0.29	23.97±0.40	73955.43±14826.73	15769.10±4170.90	None
#45	M	18.51±0.38	24.22±0.29	48770.07±11320.14	13323.92±2551.12	None
#46	F	17.20±0.40	24.03±0.49	116770.19±29525.91	15340.46±4653.37	m.3460G>A $^{+/-}$

#47	M	17.32±0.09	24.12±0.26	105524.45±6445.53	14161.57±2213.04	m.11778 G>A$^{+/-}$
#48	M	17.84±0.41	24.69±0.17	76591.06±22458.17	9824.90±1034.44	m.14484 T>C$^{+/-}$
Mean Value		17.78±0.68	24.20±0.75	84008.00±29543.54	14584.29±5334.32	N/A

Note: M, male; F, Female; CN, copy number; None: No three mtDNA mutations; Red highlighted # 25 is not included in the analysis as a discrete value; $^{+/-}$, Heteroplasmic mutation.

Supplemental Table 7. Comparison of the quantitative results from triplex-probe qPCR and SYBR Green qPCR

Sample ID#	mtDNA[1] (12s rRNA)	*Wt* (CN±SD) [2]			*Mut* (CN±SD) [2]		
		(m.3460G>A)	(m.11778G>A)	(m.14484T>C)	(m.3460G>A)	(m.11778G>A)	(m.14484T>C)
#1	98322.07 ±20603.91	89439.12 ±11101.95	90739.29 ±18107.79	106729.81 ±15325.24	0	0	0
#2	93883.49 ±17708.88	91551.70 ±15603.59	81917.79± 15850.30	110065.22 ±26887.46	0	0	0
#3	35654.37 ±9139.16	35282.55 ±5065.03	34213.43 ±3089.84	40135.53 ±9972.42	0	0	0
#4	93648.37 ±18128.06	98869.33 ±21412.93	93384.45 ±25056.41	105528.07 ±27196.15	0	0	0
#5	105852.31± 26836.04	97728.55 ±11023.09	101725.96 ±31382.97	116328.16 ±22712.03	0	0	0
#6	99092.26± 15008.93	97358.88 ±24456.21	98586.73 ±9731.18	104184.80 ±28890.96	0	0	0
#7	87020.46 ±24112.33	93024.51 ±18869.71	79686.09 ±1732.75	87251.70 ±10269.24	0	0	0
#8	79263.34 ±9059.29	72249.30 ±14729.59	67600.66 ±11537.44	78187.72 ±14550.81	0	0	0
#9	101593.37 ±2248.62	98137.91 ±9230.78	92862.41 ±7221.31	105777.33 ±24424.50	0	0	0
#10	107251.53 ±23734.86	101474.61 ±27950.21	106932.16 ±22729.98	112389.76 ±1360.83	0	0	0
#11	86277.68 ±5868.68	75835.10 ±15912.61	90612.66 ±23256.17	86371.75 ±12027.87	0	0	0
#12	125582.32 ±33983.05	115998.15 ±6201.33	124822.97 ±32253.96	138540.64 ±42203.93	0	0	0
#13	129546.68 ±33882.27	133556.28 ±31001.17	135028.07 ±30198.99	138589.20 ±23455.67	0	0	0
#14	102218.66 ±9505.80	95245.64 ±5800.42	103261.67 ±24160.96	110180.89 ±20812.93	0	0	0
#15	124383.68	113120.35	111379.19	121996.14	0	0	0

	±31882.47	±21924.31	±14830.79	±18314.55			
#16	113267.22 ±32726.05	110462.06 ±14177.97	102587.97 ±22714.75	115163.92 ±12310.51	0	0	0
#17	125630.57 ±35120.80	116199.29 ±14969.13	121832.4 ±33570.66	126810.75 ±14509.38	0	0	0
#18	99773.48 ±26976.03	86253.54 ±10737.69	102861.09 ±21812.31	96715.80 ±22319.12	0	0	0
#19	77266.79 ±11628.61	76194.19 ±11882.74	71721.99 ±2161.07	75936.41 ±16373.24	0	0	0
#20	88413.47 ±19758.86	84953.59 ±13753.96	76888.68 ±10957.34	91952.75 ±28104.46	0	0	0
#21	15456.09 ±4354.33	10621.08 ±2189.35	9065.16 ±2607.89	16182.46 ±2852.55	0	0	0
#22	38085.59 ±10935.92	31195.65 ±4819.81	33908.09 ±5613.44	42137.23 ±4070.16	0	0	0
#23	85804.71 ±3327.53	74276.70 ±14109.67	85873.36 ±19769.22	89479.49 ±12807.81	0	0	0
#24	115251.77 ±33186.12	107137.90 ±22721.89	105384.69 ±18568.59	120749.91 ±28585.00	0	0	0
#25	58884.23 ±13172.47	55255.89 ±2517.51	50154.04 ±9597.48	57262.34 ±8107.67	0	0	0
#26	86070.55 ±3554.65	85426.17 ±11548.05	77734.76 ±9152.77	90629.48 ±6141.03	0	0	0
#27	82658.82 ±21650.74	77489.99 ±13660.29	74067.4 ±10547.5	85195.81 ±10363.95	0	0	0
#28	92494.52 ±26637.88	81781.48 ±8718.59	86064.10 ±17693.78	98194.76 ±7606.25	0	0	0
#29	87480.79 ±5603.67	82408.90 ±10287.99	79241.45 ±15817.74	92208.02 ±10390.06	0	0	0
#30	86455.23 ±14021.64	82046.92 ±25221.90	89274.26 ±28552.34	97303.43 ±25896.87	0	0	0
#31	60393.82 ±4832.23	55215.52 ±10211.75	53061.93 ±7334.99	61728.49 ±9463.76	0	0	0

#32	73025.19 ±12727.79	69825.36 ±10423.77	71446.35 ±21487.70	78246.28 ±19231.60	0	0	0
#33	83660.36 ±18202.10	82077.15 ±11907.91	90522.7 ±15892.84	87618.69 ±2375.72	0	0	0
#34	77607.16 ±24793.95	73279.91 ±3358.61	68986.86 ±10175.96	92719.29 ±19380.08	0	0	0
#35	90575.87 ±21207.73	82279.92 ±12403.21	86815.93 ±11332.03	89488.73 ±24888.58	0	0	0
#36	88413.47 ±26423.63	88622.86 ±20041.06	90420.99 ±29243.82	88673.07 ±2449.15	0	0	0
#37	17355.46 ±5165.23	18291.14 ±2818.58	14597.00 ±4155.6	22100.00 ±3675.74	0	0	0
#38	79482.12 ±24697.71	76371.26 ±12946.99	75426.72 ±11728.73	86444.18 ±29068.21	0	0	0
#39	53172.78 ±13540.97	48804.42 ±7996.00	50533.89 ±4974.76	61519.62 ±15737.04	0	0	0
#40	63423.13 ±9913.33	59408.95 ±5242.08	61605.20 ±10069.48	61108.16 ±11546.32	0	0	0
#41	69942.09 ±9592.96	59927.30 ±14574.88	58807.79 ±11813.17	78599.76 ±20252.84	0	0	0
#42	63047.64 ±10031.43	51477.63 ±9298.20	62451.49 ±14285.09	64923.71 ±14604.89	0	0	0
#43	38085.59 ±11108.77	34883.61 ±2657.38	34627.12 ±7105.35	43640.55 ±8936.42	0	0	0
#44	89508.30 ±24194.9	89714.04 ±10425.18	88346.33 ±9718.22	97997.95 ±23608.38	0	0	0
#45	76393.72 ±14798.73	78771.44 ±24731.87	79224.48 ±7539.36	83498.21 ±15139.36	0	0	0
#46	86230.63 ±26993.65	44499.62 ±8522.29	86934.62 ±24844.35	92273.64 ±6660.46	42867.40 ±6678.75	0	0
#47	81453.37 ±12837.61	73383.73 ±3914.34	37667.51 ±5666.63	88623.24 ±9441.24	0	353024.09 ±40501.70	0
#48	56773.20	50278.21	53000.73	39322.17	0	0	16045.38

	±6000.66	±6059.20	±8113.36	±934.46			±3270.40
Mean	83239.24<br>±25982.15	77243.49<br>±28414.64	77997.72<br>±30458.59	87014.69<br>±31163.90	42867.40<br>±6678.75	353024.09<br>±40501.70	16045.38<br>±3270.40

Note: CN, Copy number; [1] SYBR Green PCR; [2] TaqMan-MGB probe qPCR

Supplemental Table 8. The capability of the triplex-probe qPCR in distinguishing different contents of three mtDNA mutations

Mutation content Interval	Sample ID# [1]	Mutation content (%)	Detected mutation content (%) ± SD		
			m. 3460G>A	m.11778G>A	m.14484T>C
0~25%	#05	10	15.15±0.16	08.02±0.24	08.72±0.01
	#07	15	23.38±0.31	16.53±0.15	12.65±0.03
	#13	20	29.61±0.03	21.98±0.03	18.08±0.13
25~80%	#01	30	39.32±0.16	31.16±0.71	26.95±0.32
	#02	35	42.71±0.41	33.05±0.87	33.36±0.30
	#04	40	47.00±0.27	37.03±0.20	36.00±0.85
	#08	45	48.29±0.57	41.54±0.92	39.20±0.17
	#09	50	50.14±0.15	43.19±1.08	42.13±0.21
	#11	55	63.69±0.53	59.79±0.65	59.50±0.42
	#12	60	65.70±0.61	63.32±0.96	59.79±0.86
	#14	65	68.34±0.31	64.72±0.85	66.15±1.02
	#15	70	70.95±0.03	66.86±0.64	72.98±1.02
	#16	75	74.54±0.51	71.96±0.73	77.59±0.49
80~100%	#03	85	81.08±0.27	81.26±0.06	82.82±0.07
	#06	90	84.10±0.53	86.11±0.57	89.79±0.28
	#10	95	89.01±0.37	89.92±0.09	96.80±0.10

Note: Mutation content: Copy number ratio of mutant mtDNA to total mtDNA; [1] Sample ID# means the order number of different mixture samples by mixing standard plasmid and genomic DNA.

Full-length gels of representative T-ARMS-PCR

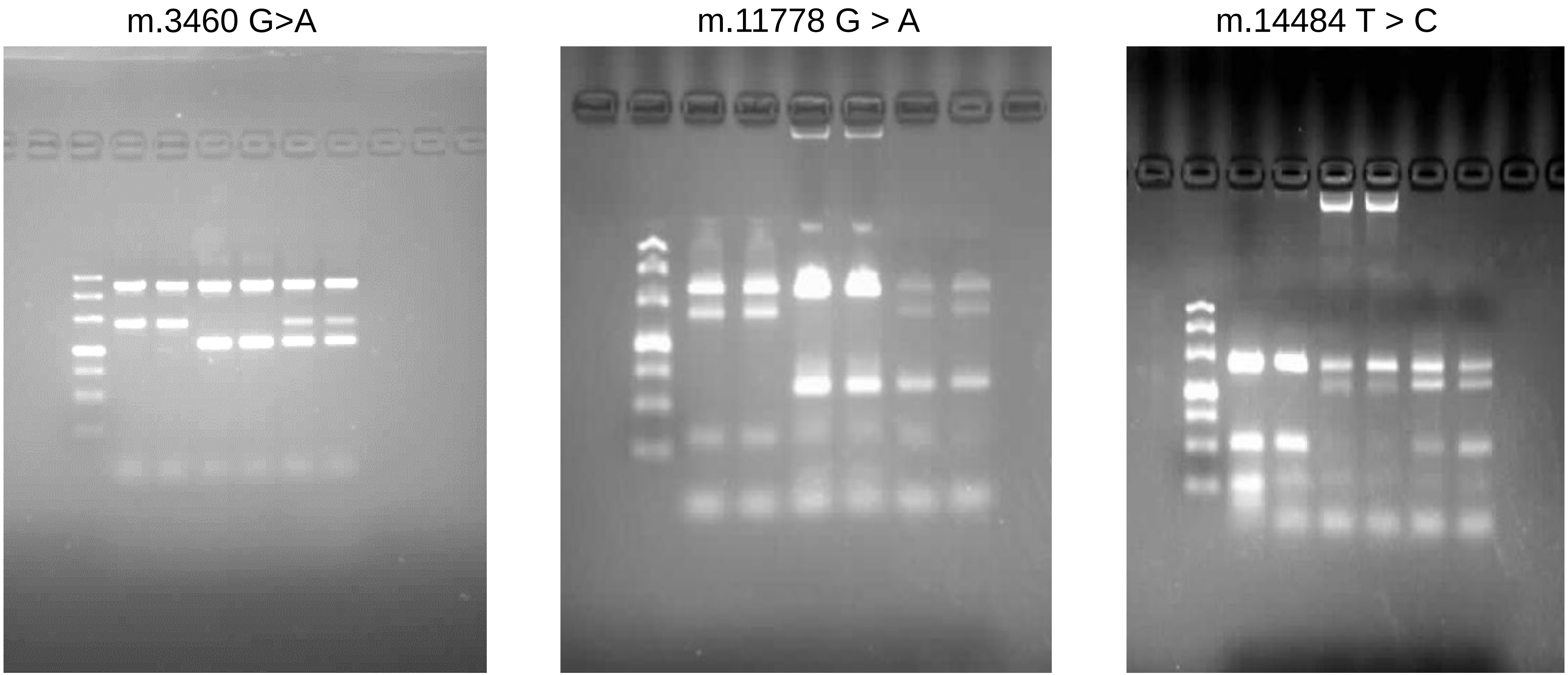

Full-length gels of sensitivity analysis

m.3460 G/G

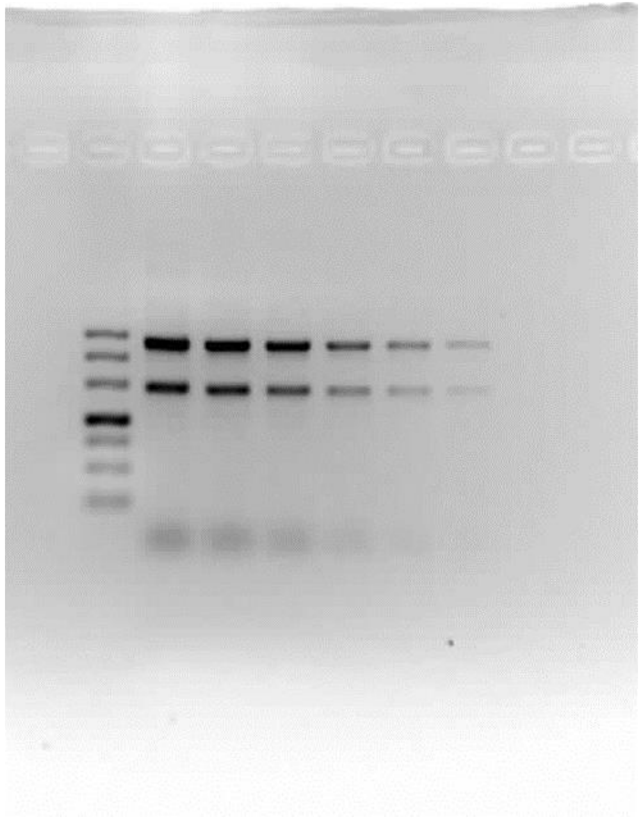

m.3460 A/A

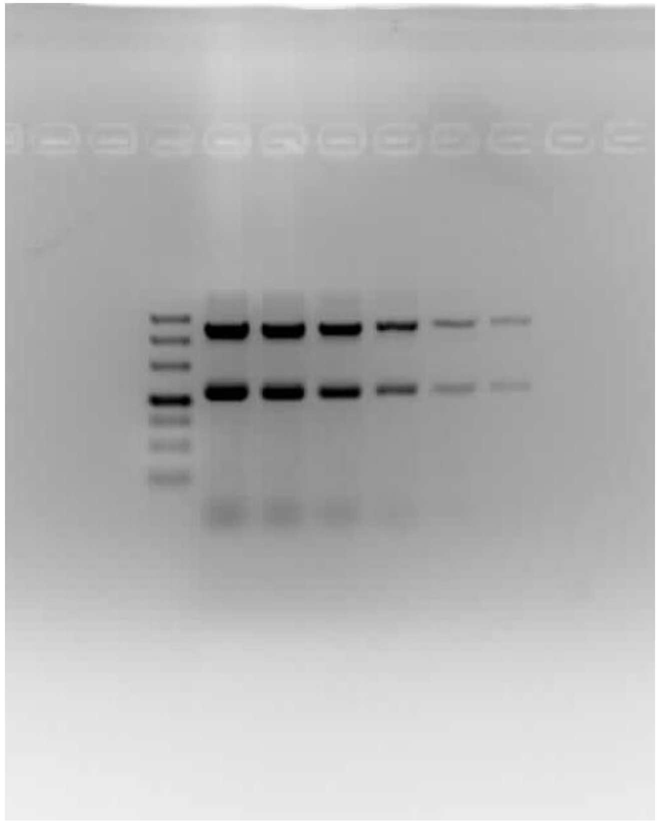

m.3460 A/G

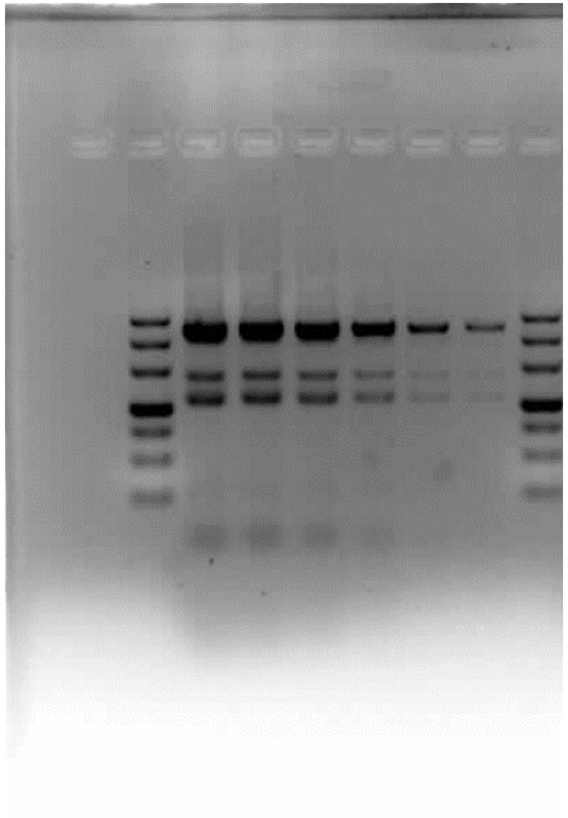

Full-length gels of sensitivity analysis

m.11778 G/G

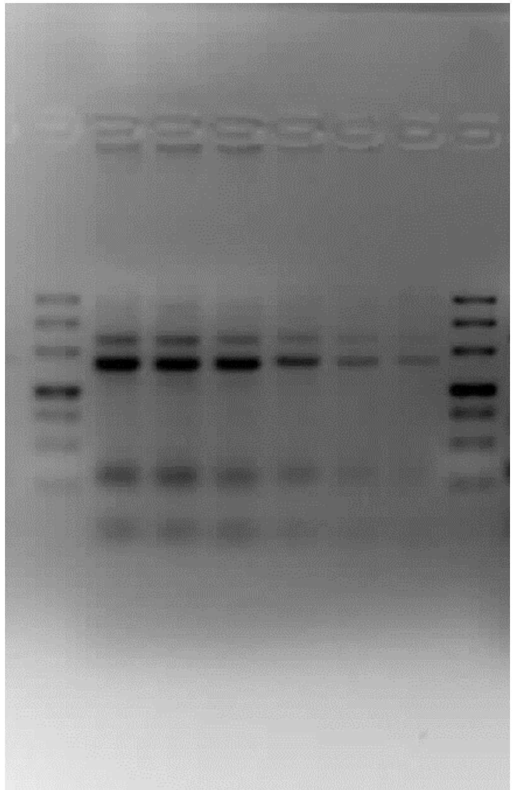

m.11778 A/A

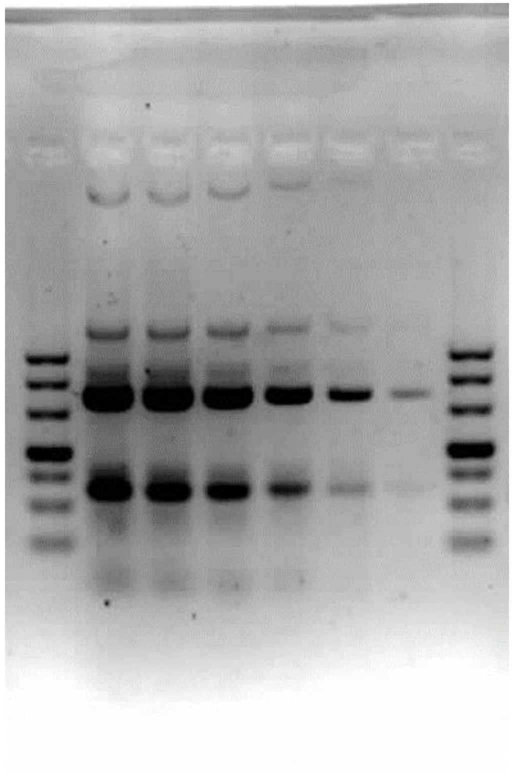

m.11778 A/G

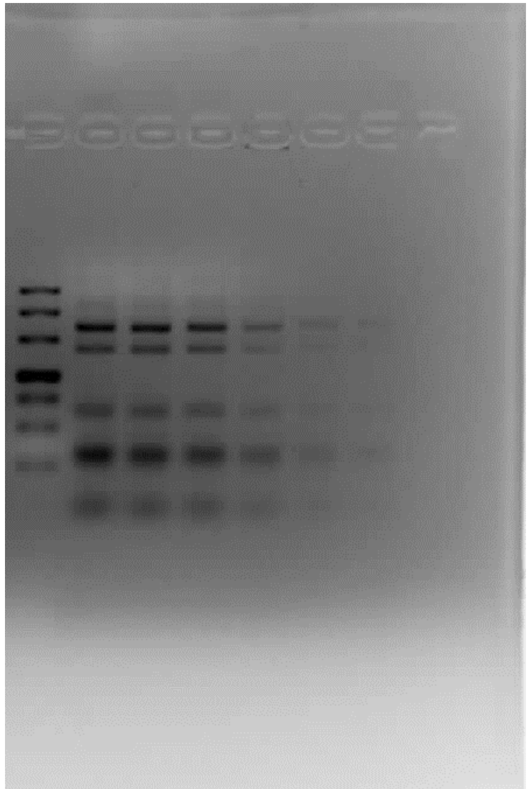

Full-length gels of sensitivity analysis

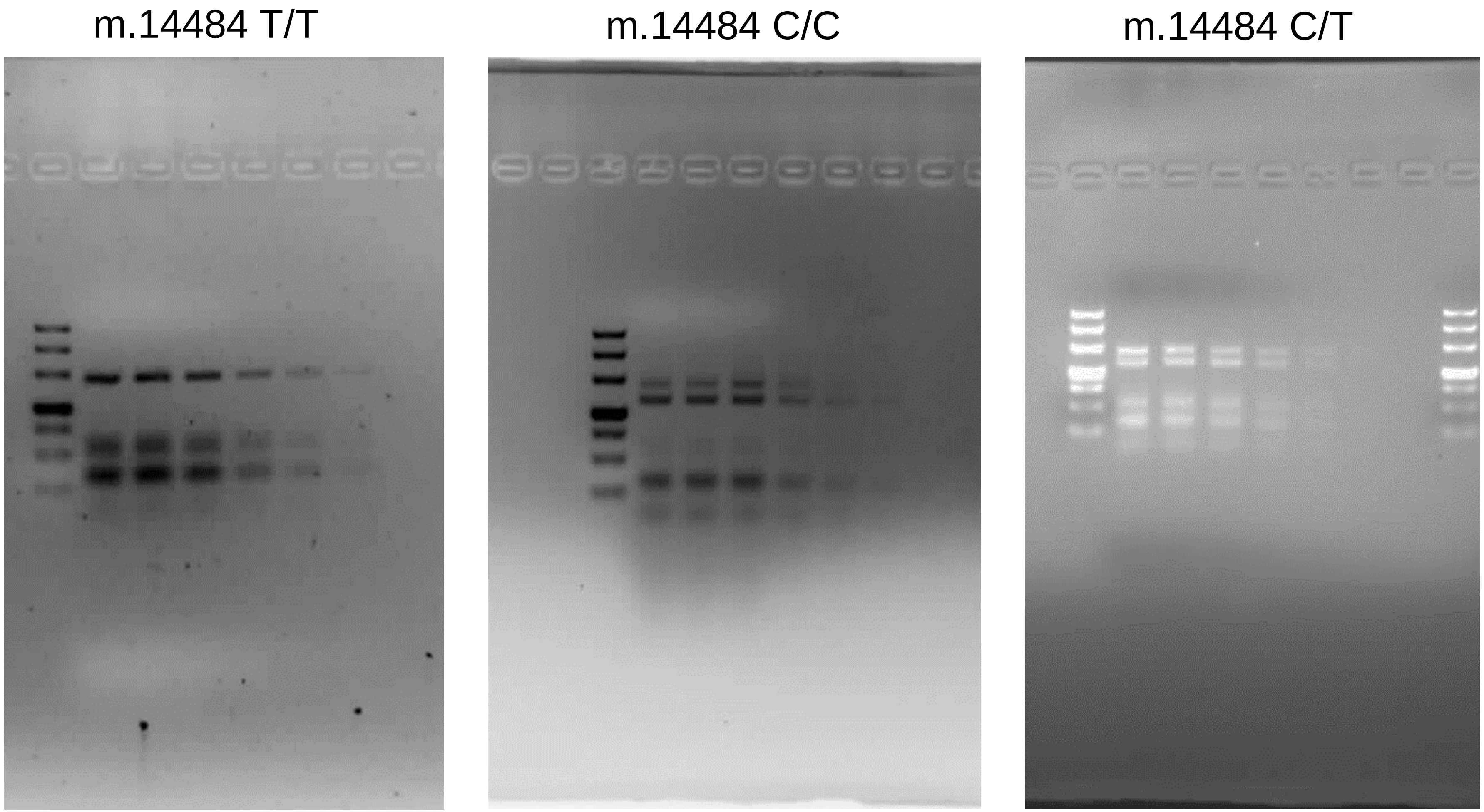

Supplement: Supplementary file 1 — Supplementary information. [file 41598_2020_69220_MOESM1_ESM.pdf]
